# Incorporating prior knowledge induced from stochastic differential equations in the classification of stochastic observations

**DOI:** 10.1186/s13637-016-0036-y

**Published:** 2016-01-20

**Authors:** Amin Zollanvari, Edward R. Dougherty

**Affiliations:** 1 Department of Electrical and Electronic Engineering, Nazarbayev University, Astana, 010000 Kazakhstan; 2 The Center for Bioinformatics and Genomic Systems Engineering and the Department of Electrical and Computer Engineering, Texas A&M University, College Station, 77840 Texas

**Keywords:** Classification, Gaussian processes, Stochastic differential equations, Optimal Bayesian classifier

## Abstract

In classification, prior knowledge is incorporated in a Bayesian framework by assuming that the feature-label distribution belongs to an uncertainty class of feature-label distributions governed by a prior distribution. A posterior distribution is then derived from the prior and the sample data. An optimal Bayesian classifier (OBC) minimizes the expected misclassification error relative to the posterior distribution. From an application perspective, prior construction is critical. The prior distribution is formed by mapping a set of mathematical relations among the features and labels, the prior knowledge, into a distribution governing the probability mass across the uncertainty class. In this paper, we consider prior knowledge in the form of stochastic differential equations (SDEs). We consider a vector SDE in integral form involving a drift vector and dispersion matrix. Having constructed the prior, we develop the optimal Bayesian classifier between two models and examine, via synthetic experiments, the effects of uncertainty in the drift vector and dispersion matrix. We apply the theory to a set of SDEs for the purpose of differentiating the evolutionary history between two species.

**Electronic supplementary material:**

The online version of this article (doi:10.1186/s13637-016-0036-y) contains supplementary material, which is available to authorized users.

## Introduction

A purely data-driven classifier design with small samples encounters a fundamental conundrum: since the error rate of a classifier quantifies its predictive accuracy, the salient epistemic attribute of any classifier and re-sampling strategies such as cross-validation and bootstrap is generally very inaccurate on small samples due to excessive variance and lack of regression with the true error [1]. The inability to satisfactorily estimate the error with model-free methods with small samples implies that classifier error estimation is virtually impossible without the use of prior information. Prior knowledge can be incorporated in a Bayesian framework by assuming that the feature-label distribution belongs to an uncertainty class of feature-label distributions governed by a prior distribution [2, 3]. Given the latter, in conjunction with sample data, one can optimally estimate the error of any classifier, relative to the mean square error (MSE) between the true and estimated errors, where expectations are taken with respect to a posterior distribution derived from the prior distribution and the data [4, 5]. Hence, optimality is with respect to our prior knowledge and the data. Furthermore, one can derive an optimal classifier relative to the expected error of the classifier over the posterior distribution, this being called the *optimal Bayesian classifier* (OBC) [6, 7]. Closed-form solutions have been developed for multinomial and Gaussian models. In other situations, Markov Chain Monte Carlo (MCMC) methods can be used [8].

Having developed the statistical theory, one is confronted with an engineering problem: transform scientific knowledge given in some mathematical form into a prior distribution. Intuitively, given a set of mathematical relations among the features and labels, these relations constrain the uncertainty class of feature-label distributions that could potentially govern the classification and the relative strengths of the relations can be transformed so as to determine the probability mass of the prior distribution. For instance, in phenotype classification based on gene expression, genetic regulatory pathways constitute graphical prior knowledge and this prior knowledge can be employed to formulate a prior distribution governing the uncertainty class of feature-label distributions [9, 10]. Another genomic application involves using prior knowledge concerning RNA-seq data to form sequence-based classifiers [8].

From a general perspective, when using Bayesian methods, prior construction constitutes the highest hurdle. A half century ago, E. T. Jaynes remarked,

Bayesian methods, for all their advantages, will not be entirely satisfactory until we face the problem of finding the prior probability squarely [11].

The aim of this paper is to utilize prior knowledge in the form of stochastic differential equations (SDEs) to classify time-series data. Although we will confine ourselves to a Gaussian problem so that we can take advantage of existing closed-form OBC representations, one can envision further applications using MCMC methods. Hence, the approach taken in the present paper may lead to utilizing SDEs across a number of time-series classification problems, keeping in mind that SDEs play a major role in many disciplines including physics, biology, finance, and chemistry. Vector SDEs, our concern here, have various applications. Not only do they arise naturally in many systems with vector value states, but they also arise in many systems where the process is restricted to lie on certain manifolds [12].

In the stochastic setting, training data are collected over time processes. Given certain Gaussian assumptions, classification in the SDE setting takes the same form as ordinary classification in the Gaussian model and we can apply the optimal Bayesian classification theory once we have a prior distribution constructed in accordance with known stochastic equations. In this paper, we provide the mathematical framework to synthesize an OBC in the presence of prior knowledge induced in the form of SDEs governing the dynamics of the system. We consider a vector SDE in integral form involving a drift vector and dispersion matrix, develop the OBC between two models, and examine via synthetic experiments the effects of uncertainty in the drift vector and dispersion matrix.

We compare the performance of the OBC with quadratic discriminant analysis (QDA), a classical approach to building classifiers in the Gaussian model (see Additional file 2: Section I for definition of QDA). Such comparisons are useful because, even though the OBC is optimal given the uncertainty, its optimality is *on average* across the uncertainty class, so that its performance advantage varies for different feature-label distributions in the uncertainty class (and can be disadvantageous for some distributions, although these will have small probability mass in the posterior distribution). Comparison to QDA is instructive because, as we will explain in the next section, QDA is a sample-based approximation to the optimal classifier for the true feature-label distribution. In addition to synthetic experiments, we apply optimal Bayesian classification using a form of the Ornstein-Uhlenbeck process that has been employed for modeling the evolutionary change of species; specifically, we use a set of SDEs to construct a classifier to differentiate the evolutionary history between two species.

## Background

### Classification

In a two-class classification, the population is characterized by a feature-label distribution *F* for a random pair (**X**,*Y*), where **X** is a vector of *p* features and *Y* is the binary label, 0 or 1, of the class containing **X**. The *prior class probabilities* are defined by *c*_*j*_=*P*(*Y*=*j*) and the *class-conditional densities* by *p*_*j*_(**x**)=*p*(**x**∣*Y*=*j*), for *j*=0,1. To avoid trivialities, we assume min{*c*_0_,*c*_1_}≠0. A *classifier* is a function *ψ*(**X**) assigning a binary label to each feature vector **X**. The error, *ε*[*ψ*], of *ψ* is the probability *P*(*ψ*(**X**)≠*Y*), which can be decomposed into *ε*=*c*_0_ *ε* ^0^+*c*_1_ *ε* ^1^, where *ε* ^*j*^=*P*(*ψ*(**X**)=1−*j*|*Y*=*j*), for *j*=0,1. A classifier with minimum error among all classifiers is known as a *Bayes classifier* for *F*. The minimum error is called the *Bayes error*. Epistemologically, the error is the key issue since it quantifies the predictive capacity.

In practice, *F* is unknown and a *classification rule* *ψ* is used to design a classifier *ψ*_*n*_ from a random sample *S*_*n*_={(**X**_1_,*Y*_1_),(**X**_2_,*Y*_2_),…,(**X**_*n*_,*Y*_*n*_)} of pairs drawn from *F*. If feature selection is involved, then it is part of the classification rule. Since the true classifier error *ε*[ *ψ*_*n*_] depends on *F*, which is unknown, *ε*[*ψ*_*n*_] is unknown. The true error must be estimated by an *estimation rule*, *Ξ*. Thus, the random sample *S*_*n*_ yields a classifier *ψ*_*n*_=*Ψ*(*S*_*n*_) and an error estimate $\hat {\varepsilon } [\!\psi _{n}]=\Xi (S_{n})$ (see Additional file 2: Section II for more information).

When a large amount of data is available, the sample can be split into independent training and test sets, the classifier being designed on the training data and its error being estimated by the proportion of errors on the test data; however, when data are limited, the sample cannot be split without leaving too little data to design a good classifier. Hence, training and error estimation must take place on the same data set. As noted in Section 1, accurate error estimation is virtually impossible with small samples in the absence of distributional assumptions.

### Optimal Bayesian classification

Distributional assumptions can be imposed by defining a prior distribution over an uncertainty class of feature-label distributions. This results in a Bayesian approach with the uncertainty class being given a prior distribution and the data being used to construct a posterior distribution.

Let *Π*_0_ and *Π*_1_ denote the class-0 and class-1 conditional distributions, respectively; let *c* be the probability of a point coming from *Π*_0_ (the “mixing” probability); and let *Π*_0_ and *Π*_1_ be parameterized by *θ*_0_ and *θ*_1_, respectively. The overall model is parameterized by *θ*=(*c*,*θ*_0_,*θ*_1_) with prior distribution *π*(*θ*). Given a random sample, *S*_*n*_, a classifier *ψ*_*n*_ is designed and we wish to minimize the MSE between its true error, *ε*, and an error estimate, $\widehat {\varepsilon }$. The minimum mean square error (MMSE) error estimator is the expected true error, $\widehat {\varepsilon }(\psi _{n},S_{n})=\mathrm {E}_{\theta }[\varepsilon (\psi _{n},\theta)|S_{n}]$. The expectation given the sample is over the posterior density of *θ*, denoted by *π* ^∗^(*θ*). Thus, we write the Bayesian MMSE error estimator as $\widehat {\varepsilon }=\mathrm {E}_{\pi ^{\ast }}[\varepsilon ]$.

The Bayesian error estimate is not guaranteed to be the optimal error estimate for any particular feature-label distribution but optimal for a given sample, and assuming the parameterized model and prior probabilities, it is both optimal on average with respect to MSE and unbiased when averaged over all parameters and samples. To facilitate analytic representations, we assume *c*, *θ*_0_, and *θ*_1_ are all mutually independent prior to observing the data. Denote the marginal priors of *c*, *θ*_0_, and *θ*_1_ by *π*(*c*), *π*(*θ*_0_), and *π*(*θ*_1_), respectively, and suppose data are used to find each posterior, *π* ^∗^(*c*), *π* ^∗^(*θ*_0_), and *π* ^∗^(*θ*_1_), respectively. Independence is preserved, i.e., *π* ^∗^(*c*,*θ*_0_,*θ*_1_)=*π* ^∗^(*c*)*π* ^∗^(*θ*_0_)*π* ^∗^(*θ*_1_) [4].

If *ψ*_*n*_ is a trained classifier given by *ψ*_*n*_(**x**)=0 if **x**∈*R*_0_ and *ψ*_*n*_(**x**)=1 if **x**∈*R*_1_, where *R*_0_ and *R*_1_ are measurable sets partitioning the sample space, then the Bayesian MMSE error estimator can be found from *effective class-conditional densities*, which are derived by taking the expectations of the individual class-conditional densities with respect to the posterior distribution,

(1) $$ f\left(\mathbf{x}|y\right) =\int_{\mathbf{\Theta }_{y}}f_{\theta_{y}}\left(\mathbf{x}|y\right) \pi^{\ast }\left(\theta_{y}\right) d\theta_{y}.  $$

Using these [6] (see Additional file 2: Section III for more information),

(2) $$ {}\widehat{\varepsilon }\left(\psi_{n},S_{n}\right) \,=\,\mathrm{E}_{\pi^{\ast}}[\!c]\!\int_{R_{1}}\!f\!\left(\mathbf{x}|0\right) d\mathbf{x}+(1-\mathrm{E}_{\pi^{\ast }}[\!c]\!)\int_{R_{0}}f\!\left(\mathbf{x}|1\right) d\mathbf{x}.   $$

In the context of a prior distribution, an optimal Bayesian classifier, *ψ*_OBC_, is any classifier satisfying

(3) $$ \mathrm{E}_{\pi^{\ast }}\left[ \varepsilon (\psi_{\text{OBC}},\theta) \right] \leq \mathrm{E}_{\pi^{\ast }}\left[ \varepsilon (\psi,\theta) \right]   $$

for all $\psi \in \mathcal {C}$, where $\mathcal {C}$ is an arbitrary family of classifiers. Under the Bayesian framework, this is equivalent to minimizing the probability of error,

(4) $$ \begin{aligned} \mathrm{P}\left(\psi_{n}\left(\mathbf{X}\right) \neq Y|S_{n}\right)& =\mathrm{E}_{\pi^{\ast }}\left[ P\left(\psi_{n}\left(\mathbf{X}\right) \neq Y|\theta,S_{n}\right) \right]\\ & =\widehat{\varepsilon }\left(\psi_{n},S_{n}\right). \end{aligned}  $$

If $\mathcal {C}$ is the set of all classifiers with measurable decision regions (which we will assume), then an optimal Bayesian classifier, *ψ*_OBC_, satisfying (3) for all $\psi \in \mathcal {C}$ exists and is given pointwise by [6]

(5) $$ \psi_{\text{OBC}}\left(\mathbf{x}\right) =\left\{\! \begin{array}{ll} 0 & \text{if }\mathrm{E}_{\pi^{\ast }}[\!c]f\left(\mathbf{x}|0\right) \geq (1-\mathrm{E}_{\pi^{\ast }}[\!c])f\left(\mathbf{x}|1\right), \\ 1 & \text{otherwise}. \end{array} \right.   $$

In many applications, especially in biomedicine, the sample *S*_*n*_ is obtained by first deciding how many sample points will be taken from each class and then randomly sampling from each class separately, the resulting sample said to be “separately sampled.” With separate sampling, the data cannot be used to generate a posterior distribution for *c*, so that *c* must be known. Stratified sampling is a special case of separate sampling in which the sample is drawn so that the proportion of sample points from class 0 is equal to *c*. In such a case, there is no posterior $\mathrm {E}_{\pi ^{\ast }\phantom {\dot {i}\!}}[\!c]$ and $\mathrm {E}_{\pi ^{\ast }\phantom {\dot {i}\!}}[\!c]$ is replaced by *c* in (5). We will utilize stratified sampling in our examples.

## Binary classification of Gaussian processes

In this section, we frame the setting in which we are working and then define the problem of binary classification in the context of Gaussian processes. To begin with, a collection {**X**_*t*_:*t*∈**T**} of $\mathbb {R}^{p}$-valued random variables defined on a common probability space $(\Omega,\mathcal {F},P)$ indexed by a parameter $ t\in \mathbf {T}\subset \mathbb {R}$ (here assumed to be time) and $\mathcal {F}$ being a *σ*-algebra of subsets of the sample space *Ω* (events) constitutes a stochastic process **X** with state space $\mathbb {R} ^{p}$. Throughout this work, we consider $\mathcal {F}$ as the *σ*-algebra of Borel subsets of $\mathbb {R}^{p}$. A stochastic process **X** is *adapted* to an increasing family of *σ*-algebra $\{\mathcal {F}_{t}:t\geq 0\}$ (a filtration) if for each *t*≥0, **X**_*t*_ is $\mathcal {F}_{t}$-measurable.

We study classification in the context of multivariate Gaussian processes (see Additional file 2: Section IV for a review of literature pertaining to classification of stochastic processes). Consider the *p*-dimensional column random vectors $\mathbf {X}_{t_{1}}$, $\mathbf {X}_{t_{2}}$,...., $\mathbf {X}_{t_{N}}$. A random process **X** is a multivariate Gaussian process if any finite-dimensional vector $\left [\mathbf {X}_{t_{1}}^{T},\mathbf {X}_{t_{2}}^{T},...,\mathbf {X}_{t_{N}}^{T}\right ]^{T} $ possesses a multivariate normal distribution $\mathcal {N}\left (\boldsymbol {\mu }_{\mathbf {t}_{N}},\boldsymbol {\Sigma }_{\mathbf {t}_{N}}\right)$, where

(6) $$ \boldsymbol{\mu }_{\mathbf{t}_{N}}=\left[\boldsymbol{\mu }_{t_{1}}^{T}, \boldsymbol{\mu }_{t_{2}}^{T},...,\boldsymbol{\mu }_{t_{N}}^{T}\right]_{Np\times 1}^{T},   $$

with $\boldsymbol {\mu }_{{t}_{i}\phantom {\dot {i}\!}}=E[\mathbf {X}_{t_{i}\phantom {\dot {i}\!}}]$, and $\boldsymbol { \Sigma }_{\mathbf {t}_{N}\phantom {\dot {i}\!}}$ is the *N* *p*×*N* *p* covariance matrix dependent on **t**_*N*_=[*t*_1_,*t*_2_,...,*t*_*N*_]^*T*^ and structured as

(7) $$ \begin{aligned} \boldsymbol{\Sigma }_{\mathbf{t}_{N}}\,=\, \left[ \begin{array}{cccc} \boldsymbol{\Sigma }_{{t}_{1},{t}_{1}} &\boldsymbol{\Sigma }_{t_{1},{t}_{2}} &... & \boldsymbol{\Sigma }_{t_{1},{t}_{N}} \\[1ex] \boldsymbol{\Sigma }_{{t}_{2},{t}_{1}} &\boldsymbol{\Sigma }_{t_{2},{t}_{2}} &... & \boldsymbol{\Sigma }_{t_{2},{t}_{N}} \\... &... &... &... \\ \boldsymbol{\Sigma }_{{t}_{N},{t}_{1}} & \boldsymbol{\Sigma }_{t_{N},{t}_{2}} &... &\boldsymbol{\Sigma }_{t_{N},{t}_{N}} \end{array} \right]_{Np\times Np}, \end{aligned}   $$

where

(8) $$ \boldsymbol{\Sigma }_{{t}_{i},{t}_{j}}=E\left[\left(\mathbf{X}_{t_{i}}-E(\mathbf{X} _{t_{i}}))(\mathbf{X}_{t_{j}}-E(\mathbf{X}_{t_{j}})^{T}\right)\right].   $$

We refer to **t**_*N*_ as the *observation time vector*. For any fixed *ω*∈*Ω*, a *sample path* is a collection {**X**_*t*_(*ω*):*t*∈**t**}. We denote a realization of **X** at sample path *ω* and time vector **t**_*N*_ by $ \mathbf {x}_{\mathbf {t}_{N}\phantom {\dot {i}\!}}(\omega)$.

We consider a general framework, referred to as *binary classification of Gaussian processes (BCGP)*. Consider two independent multivariate Gaussian processes **X** ^0^ and **X** ^1^, where for any **t**_*N*_, **X** ^0^ and **X** ^1^ possess mean and covariance $\boldsymbol {\mu }_{\mathbf {t}_{N}}^{0}$ and $\boldsymbol {\Sigma }_{\mathbf {t}_{N}}^{0}$, and $\boldsymbol {\mu }_{\mathbf {t}_{N}}^{1}$ and $\boldsymbol {\Sigma }_{\mathbf {t}_{N}}^{1}$, respectively. For *y*=0,1, $ \boldsymbol {\mu }_{\mathbf {t}_{N}}^{y}$ is defined similarly to (6) with $\boldsymbol {\mu }_{{t}_{i}}^{y}=E\left [\mathbf {X}_{t_{i}}^{y}\right ]$ and $\boldsymbol {\Sigma }_{\mathbf {t}_{N}}^{y}$ is defined similarly to (7) with

(9) $$ \boldsymbol{\Sigma}_{{t}_{i},{t}_{j}}^{y}=E\left[\left(\mathbf{X}_{t_{i}}^{y}-E(\mathbf{X}_{t_{i}}^{y})\right)\left(\mathbf{X}_{t_{j}}^{y}-E(\mathbf{X} _{t_{j}}^{y})\right)^{T}\right].   $$

Let $\mathbf {S}_{\mathbf {t}_{N}}^{y}$ denote a set of *n* ^*y*^ *sample paths* from process **X** ^*y*^ at **t**_*N*_,

(10) $$ \mathbf{S}_{\mathbf{t}_{N}}^{y}=\left\{\mathbf{x}_{\mathbf{t}_{N}}^{y}(\omega_{1}),\mathbf{x}_{\mathbf{t}_{N}}^{y}(\omega_{2}),\ldots,\mathbf{x}_{ \mathbf{t}_{N}}^{y}(\omega_{n^{y}})\right\}.  $$

We assume that **t**_*N*_ is the same for both classes. Let $\mathbf {X}_{\mathbf {t}_{N}}^{y}(\omega _{s})$ denote a future test sample path observed on the same observation time vector as the training sample paths, where *y*∈{0,1} indicates the label of the class-conditional process the sample path is coming from, either **X** ^0^ or **X** ^1^. Note that, as compared with the classical probabilistic definition of classification where the sample points are observations of *p*-dimension, here we define stochastic-process classification in connection with a set of sample paths, which can be considered as observations of *Np* dimension. A classification problem arises from the fact that the experimenter is blind to the class label of $\mathbf {X}_{\mathbf {t}_{N}}^{y}(\omega _{s})$, i.e., to *y*, and desires a discriminant $\psi _{\mathbf {t}_{N}\phantom {\dot {i}\!}}(.)$ such that

(11) $$ y= \left\{ \begin{array}{ll} 0,\quad \text{if}\;\;\psi_{\mathbf{t}_{N}}\left(\mathbf{x}_{\mathbf{t} _{N}}^{y}(\omega_{s})\right)>0 \\ 1,\quad \text{otherwise}. \end{array} \right.\,.   $$

Other types of classification could be defined. For example, one might be interested in classifying a test sample path $\mathbf {x}_{\mathbf {t}_{N+M}}^{y}(\omega _{s})$ where the observation time vector of the test sample path is obtained by augmenting **t**_*N*_ by another vector [*t*_*N*+1_,*t*_*N*+2_,...,*t*_*N*+*M*_]_,_ where *M* is a positive integer. In this case, the time of observation for the future sample path is extended. Similarly, one may define problems where the future time of observation is shrunken to a subset of time points in **t**_*N*_ or problems where the future observation time vector is a set of time points totally or partially different from time points in **t**_*N*_. Throughout this work, we are mainly concerned with solving the classification problem as defined in (11), which we refer to as the *standard* type, and we discuss the feasibility of solving other cases when possible.

### General presentation of stochastic differential equations (SDEs)

To define SDEs, we consider a diffusion process, the most fundamental being the Wiener process. For a general definition of a *q*-dimensional Wiener process, see the Appendix. Let **W**={**W**_*t*_:*t*≥0} be a *q*-dimensional Wiener process. For each sample path and for 0≤*t*_0_≤*t*≤*T*, we consider a vector SDE in the integral form as follows:

(12) $$ \begin{aligned} \mathbf{X}_{t}(\omega)&=\mathbf{X}_{t_{0}}(\omega)+\int_{t_{0}}^{t}\mathbf{f }\left(s,\mathbf{X}_{s}(\omega)\right) ds\\ &\quad+\int_{t_{0}}^{t}\mathbf{G}\left(s,\mathbf{X}_{t}(\omega)\right) d\mathbf{W}_{s}(\omega),  \end{aligned}  $$

where $\mathbf {f}:[\!0,T]\times \Omega \rightarrow \mathbb {R}^{p}$ (the *p*-dimensional drift vector) and $\mathbf {G}:[\!0,T]\times \Omega \rightarrow \mathbb {R}^{p\times q}$ (the *p*×*q* dispersion matrix). The first integral in (12) is an ordinary Lebesgue integral, and throughout this work, we assume an Itô integration for the second integral. With slightly more work, the results can be extended to Stratonovich integration. Let $\mathcal {L}$ be the *σ*-algebra of Lebesgue subsets of $\mathbb {R}$. A function *h*(*t*,*ω*) defined on a probability space $(\Omega,\mathcal {F},P)$ belongs to $\mathcal {L}_{T}^{\omega }$ if it is jointly $\mathcal {L}\times \mathcal {F}$ measurable, *h*(*t*,.) is $\mathcal {F}_{t}$-measurable for each *t*∈[ 0,*T*], and with probability 1, ${\int _{0}^{T}}h(s,\omega)^{2}ds<\infty $. Let *f* ^*i*^ and *g* ^*i*,*j*^ denote the components of **f** and **G**, respectively. If we assume **X**_0_(*ω*) is $\mathcal {F}_{0}$-measurable and if $\sqrt {|f^{i}|}\in \mathcal {L}_{T}^{\omega }$ and $g^{i,j}\in \mathcal {L}_{T}^{\omega }$, then each component of the *p*-dimensional process **X**_*t*_(*ω*) is $\mathcal {F}_{t}$-measurable [12]. The $\mathcal {F}_{t}$-measurability of **X**_*t*_(*ω*) along with the martingale property of **W** indicates “nonanticipativeness” of **X**_*t*_(*ω*) in general.

The integral Eq. (12) is commonly written in a symbolic form as

(13) $$ \mathrm{d}\mathbf{X}_{t}=\mathbf{f}(t,\mathbf{X}_{t})\mathrm{d}t+\mathbf{G}(t, \mathbf{X}_{t})\mathrm{d}\mathbf{W}_{t},   $$

which is the representation of a vector SDE.

## SDE prior knowledge in the BCGP model

Prior knowledge in the form of a set of stochastic differential equations constrains the possible behavior of the dynamical system to an uncertainty class. If such prior knowledge is available, then it can be used in the BCGP model to improve classification performance. The core underlying assumption of the BCGP model is that the data are generated from two Gaussian processes for which binary classification is desired. In this regard, we define *valid prior knowledge* (in the form of SDEs) as a set of SDEs with a unique solution that does not contradict the Gaussianity assumption of the dynamics of the model. For nonlinear **f**(*t*,**X**_*t*_) and **G**(*t*,**X**_*t*_) (w.r.t. to state **X**_*t*_), the solution of SDE (13) is generally a non-Gaussian process. Fortunately, under a wide class of linear functions, the SDE solutions are Gaussian. To wit, the SDEs become valid prior knowledge for each class-conditional process defined in the BCGP model. Henceforth, we focus on this type of SDE.

For class label *y*=0,1, the linear classes of SDEs that we consider are defined by replacing

(14) $$ \begin{aligned} &\mathbf{f}^{y}(t,\mathbf{X}_{t})=\mathbf{A}^{y}(t)\mathbf{X}_{t}^{y}+ \mathbf{a}^{y}(t), \\ & \mathbf{G}^{y}(t,\mathbf{X}_{t})=\mathbf{B}^{y}(t), \end{aligned}   $$

in (13) with **A** ^*y*^(*t*) (a *p*×*p* matrix), **a** ^*y*^(*t*) (a *p*×1 vector), and **B** ^*y*^(*t*) (a *p*×*q* matrix), these being measurable and bounded on [ *t*_0_,*T*]. This results in

(15) $$ \begin{aligned} \mathrm{d}\mathbf{X}_{t}^{y}=\!(\mathbf{A}^{y}(t)\mathbf{X}_{t}^{y}+ \mathbf{a}^{y}(t))\mathrm{d}t+\mathbf{B}^{y}(t)\mathrm{d}\mathbf{W}_{t}^{y}, \,\,\,\mathbf{X}_{t_{0}}^{y}(\omega)\!=c^{y}. \end{aligned}   $$

This initial value problem has a unique solution that is a Gaussian stochastic process if and only if the initial conditions *c* ^*y*^ are constant or normally distributed (Theorem 8.2.10 [13]). Note that in this model, **G** ^*y*^(*t*,**X**_*t*_) (i.e. **B** ^*y*^(*t*)) is independent of *ω*. Under this model, it can be shown that the mean (at a time index *t*_*i*_) and the covariance matrix (at *t*_*i*_ and *t*_*j*_) of the Gaussian process $\mathbf {X}_{t}^{y}$ are given by [13]

(16) $$ \begin{aligned} \mathbf{m}_{t_{i}}^{y}=E\left[\!\mathbf{X}_{t_{i}}^{y}\right]=\boldsymbol{\Phi }^{y}(t_{i})\left(E[\!c^{y}]+\int_{t_{0}}^{t_{i}}\boldsymbol{\Phi }^{y}(s)^{-1}\mathbf{a}^{y}(s)\mathrm{d}s\right) \end{aligned}   $$

and

(17) $$ {\small{\begin{aligned} {}{\boldsymbol{\Psi }}_{t_{i},t_{j}}^{y}&=E\left[ \left(\mathbf{X}_{t_{i}}^{y}-E\left[\!\mathbf{X}_{t_{i}}^{y}\right]\right)\left(\mathbf{X}_{t_{j}}^{y}-E\left[\! \mathbf{X}_{t_{j}}^{y}\right]\right)^{T}\right] \\ & =\boldsymbol{\Phi }^{y}(t_{i})\left(E\left[\left(c^{y}-E[\!c^{y}]\right)\left(c^{y}-E[\!c^{y}]\right)^{T}\right]\right.\\ &\quad\left.+\int_{t_{0}}^{t_{i}}{\boldsymbol{ \Phi }^{y}(u)}^{-1}\mathbf{B}^{y}(u){\mathbf{B}^{y}(u)}^{T}\left({\boldsymbol{\Phi }^{y}(u)}^{-1}\right)^{T}\mathrm{d}u\right) {\boldsymbol{\Phi }^{y}(t_{j})}^{T}, \end{aligned}}}  $$

where *t*_0_≤*t*_*i*_≤*t*_*j*_≤*T* and ***Φ*** ^*y*^(*t*_*i*_) is the fundamental matrix of the deterministic equation

(18) $$ \dot{\mathbf{X}}_{t}^{y}=\mathbf{A}^{y}(t)\mathbf{X}_{t}^{y}.   $$

### SDEs as perfect representatives for the dynamics of class-conditional processes

If the SDE model presented in (15) could perfectly represent the dynamics of the underlying stochastic processes of the BCGP model, then there would be no need for training sample paths. To see this, note that in this case ${\boldsymbol {\mu }}_{t}^{y}$ and ${\boldsymbol {\Sigma }} _{t_{i},t_{j}}^{y}$ defined in (6) and (7) are obtained by

(19) $$ \begin{aligned} & {\boldsymbol{\mu }}_{\mathbf{t}_{N}}^{y}=\mathbf{m}_{\mathbf{t}_{N}}^{y} \\ & {\boldsymbol{\Sigma}}_{\mathbf{t}_{N}}^{y}={\boldsymbol{\Psi}}_{\mathbf{t}_{N}}^{y} \end{aligned},  $$

where

(20) $$ {\mathbf{m}}_{\mathbf{t}_{N}}^{y}=\left[{\mathbf{m}_{t_{1}}^{y\,T}},{\mathbf{m} _{t_{2}}^{y\,T}},...,{\mathbf{m}_{t_{N}}^{y\,T}}\right]_{Np\times 1}^{T}   $$

and

(21) $$ \begin{aligned} \boldsymbol{\Psi }_{\mathbf{t}_{N}}^{y}\,=\, \left[ \begin{array}{cccc} \boldsymbol{\Psi }_{{t}_{1},{t}_{1}}^{y} &\boldsymbol{\Psi }_{t_{1},{t}_{2}}^{y} &... & \boldsymbol{\Psi }_{t_{1},{t}_{N}}^{y} \\[1ex] \boldsymbol{\Psi }_{{t}_{2},{t}_{1}}^{y} &\boldsymbol{\Psi }_{t_{2},{t}_{2}}^{y} &... & \boldsymbol{\Psi }_{t_{2},{t}_{N}}^{y} \\... &... &... &... \\ \boldsymbol{\Psi }_{{t}_{N},{t}_{1}}^{y} & \boldsymbol{\Psi }_{t_{N},{t}_{2}}^{y} &... &\boldsymbol{\Psi }_{t_{N},{t}_{N}}^{y}\end{array} \right]_{Np\times Np} \end{aligned},   $$

where ${\mathbf {m}_{t_{i}}^{y\,T}}$ and $\boldsymbol {\Psi }_{{t}_{i},{t} _{j}}^{y}$ are obtained from (16) and (17), respectively. Therefore, one can obtain the exact (or at least approximately exact) values of the means and auto-covariances used to characterize the Gaussian processes involved in the BCGP model. To obtain ${\mathbf {m}_{t_{i}}^{y}}$ and $\boldsymbol {\Psi }_{{t}_{i},{t}_{j}}^{y}$, two approaches can be taken. First, one may analytically solve (18) where possible and then use numerical methods to evaluate the integrations presented in (16) and (17). For example, if **A** ^*y*^(*t*)=**A** ^*y*^, i.e., being independent of *t*, the solution of (18) is given by a matrix exponential as

(22) $$ \boldsymbol{\Phi }^{y}(t)=e^{\mathbf{A}^{y}(t-t_{0})},  $$

which can be used in (16) and (17). In general, where one may not be able to analytically solve (18), numerical methods such as the Euler-Maruyama scheme [14] can be used to directly solve for $ \mathbf {X}_{t}^{y}(\omega)$ and obtain

(23) $$ {\small{\begin{aligned} \hat{\mathbf{m}}_{\mathbf{t}_{N}}^{y}&=\frac{1}{l^{y}}\sum_{i=1}^{l^{y}} \mathbf{x}_{\mathbf{t}_{N}}^{y,\text{SDE}}(\omega_{i}), \\ \hat{{\boldsymbol{\Psi}}}_{\mathbf{t}_{N}}^{y}&\,=\,\frac{1}{l^{y}-1}\!\sum_{i=1}^{l^{y}}\!\left(\mathbf{x}_{\mathbf{t}_{N}}^{y,\text{SDE}}(\omega_{i})\,-\,\bar{\mathbf{x}}_{\mathbf{t}_{N}}^{y,\text{SDE}}\right)\!\!\left(\!\mathbf{x}_{ \mathbf{t}_{N}}^{y,\text{SDE}}(\omega_{i})\,-\,\bar{\mathbf{x}}_{\mathbf{t}_{N}}^{y,\text{SDE}}\!\right)^{\!T}\!, \end{aligned}}}  $$

where $\mathbf {x}_{\mathbf {t}_{N}}^{y,\text {SDE}}(\omega _{i}),i=1,2,...,l^{y}$, are the generated sample paths obtained from solving SDEs. Since there is no restriction on generating an arbitrary number of sample paths from $\mathbf {X}_{t}^{y}(\omega)$, one can take *l* ^*y*^>>*N* *p* to have a positive definite $ \hat {{\boldsymbol {\Psi }}}_{\mathbf {t}_{N}}^{y}$ and, at the same time, obtain an accurate estimate of the actual values of ${\mathbf {m}}_{\mathbf {t}_{N}}^{y}$ and ${{\boldsymbol {\Psi }}}_{\mathbf {t}_{N}}^{y}$. In this approach, the knowledge of (16) and (17) is used in the existence of the limits ${\lim }_{l^{y}\rightarrow \infty }\,\hat {\mathbf {m}}_{\mathbf {t}_{N}}^{y}$ and ${\lim }_{l^{y}\rightarrow \infty }\,\hat {{\boldsymbol {\Psi }}}_{\mathbf {t}_{N}}^{y}$, i.e., justifies generating more sample paths as ${\lim }_{l^{y}\rightarrow \infty }\,\hat {\mathbf {m}}_{\mathbf {t}_{N}}^{y}={\mathbf {m}}_{\mathbf {t}_{N}}^{y}$ and ${\lim }_{l^{y}\rightarrow \infty }\,\hat {{\boldsymbol {\Psi }}}_{\mathbf {t}_{N}}^{y}={{\boldsymbol {\Psi }}}_{\mathbf {t}_{N}}^{y}$.

In any case, we can assume exact (approximately exact) values of $\mathbf {m}_{t_{i}}^{0}$, $\mathbf {m}_{t_{i}}^{1}$, ${\boldsymbol {\Psi }}_{t_{i},t_{j}}^{0}$, and ${\boldsymbol {\Psi }}_{t_{i},t_{j}}^{1}$ are available. The optimal discriminant in this case is obtained by using the conventional quadratic discriminant analysis (QDA), which is now defined by using the following statistic in (11):

(24) $$  {\small{\begin{aligned} {}\psi_{\mathbf{t}_{N}}^{\text{QDA}}\left(\mathbf{x}_{\mathbf{t}_{N}}^{y}(\omega_{s})\right)&\,=\,-\frac{1}{2}\left(\mathbf{x}_{\mathbf{t}_{N}}^{y}(\omega_{s})\,-\,{\mathbf{m}}_{\mathbf{t}_{N}}^{0}\right)^{T}\!{{\boldsymbol{\Psi}}}_{\mathbf{t}_{N}}^{0\;-1}\!\left(\mathbf{x}_{\mathbf{t}_{N}}^{y}(\omega_{s})-{\mathbf{m}}_{\mathbf{t}_{N}}^{0}\right) \\ & \quad+\frac{1}{2}\left(\mathbf{x}_{\mathbf{t}_{N}}^{y}(\omega_{s})-{\mathbf{m}}_{\mathbf{t}_{N}}^{1}\right) \boldsymbol{\Psi }_{t_{N}}^{1\text{}-1}\left(\mathbf{x}_{\mathbf{t}_{N}}^{y}(\omega_{s})\,-\,{\mathbf{m}}_{\mathbf{t}_{N}}^{1}\right)\\ &\quad+\frac{1}{2}\text{log}\frac{|\boldsymbol{\Psi}_{\mathbf{t}_{N}}^{1\;-1}|}{|\boldsymbol{\Psi }_{\mathbf{t}_{N}}^{0\;-1}|}-\log \frac{\alpha_{1}}{1-\alpha_{1}}. \end{aligned}}}  $$

The use of (24) is justified by the fact that the BCGP classification reduces to differentiating independent observations of *Np* dimension generated from two multivariate Gaussian distributions. Therefore, taking the same set of machinery as in [15] results in (24). We restate that in this case where (19) holds, there is no need for utilizing the sample path measurements (training sample paths) in finding the discriminant (24). This is due to the fact that the statistical properties of a Gaussian process at **t**_*N*_ are solely determined by ${\mathbf {m}}_{\mathbf {t}_{N}}^{y}$ and ***Ψ*** **t**_*N*_ *y* and, as mentioned before, either closed-form solutions of these are available or they can be approximated element-wise with an arbitrary small error rate by generating a sufficiently large number of sample paths.

The optimal solution proposed in (24) is, in fact, a function of the observation time vector of future sample paths. Therefore, if a future sample point $\mathbf {x}_{\mathbf {t}_{L}}^{y}(\omega _{s})$ is measured at an arbitrary time vector **t**_*L*_, which can be partially or totally different from **t**_*N*_, then the optimal discriminant $\psi _{\mathbf {t}_{L}}\left (\mathbf {x}_{\mathbf {t}_{L}}^{y}(\omega _{s})\right)$ is obtained by determining the solution of SDEs at **t**_*L*_ and replacing ${\mathbf {m}}_{\mathbf {t}_{N}}^{y}$ and ${\boldsymbol {\Psi }}_{\mathbf {t}_{N}}^{y}$ with ${\mathbf {m}}_{\mathbf {t}_{L}}^{y}$ and ${ \boldsymbol {\Psi }}_{\mathbf {t}_{L}}^{y}$, respectively, in (24).

### SDEs as prior information for the dynamics of class-conditional processes

In practice, the SDEs usually do not provide complete description and are then viewed as prior knowledge concerning the underlying dynamics of the BCGP model. Since we assume that a Gaussian process governs both the dynamics of each class-conditional process (BCGP model in Section 3) and its corresponding set of SDEs (by using model (15)), incompleteness of the SDEs results from the fact that (19) does not necessarily hold. We make the following assumptions on the *nature of the prior information* to which the set of SDEs corresponding to each class give rise: (i) before observing the sample paths at an observation time vector, the SDEs characterize the only information that we have about the system and (ii) the statistical properties of all Gaussian processes that may generate the data are on average (over the parameter space) equivalent to the statistical properties determined from the SDEs. The latter statement will subsequently be formalized.

Assume that the parameters $\boldsymbol {\mu }_{\mathbf {t}_{N}}^{y}$ and $\boldsymbol {\Sigma }_{\mathbf {t}_{N}}^{y}$ defining the BCGP model constitute a realization of the random vector $\mathbf {\theta }_{\mathbf {t}_{N}}^{y}=\left [\boldsymbol {\mu }_{\mathbf {t}_{N}}^{y},\boldsymbol {\Sigma }_{\mathbf {t}_{N}}^{y}\right ]$, where $\mathbf {\theta }_{\mathbf {t}_{N}}^{y}$ has a prior distribution $\pi (\mathbf {\theta }_{\mathbf {t}_{N}}^{y})$ parameterized by a set $\left \{\breve {\mathbf {m}}_{\mathbf {t}_{N}}^{y},\breve {\boldsymbol {\Psi }}_{\mathbf {t}_{N}}^{y},\nu _{\mathbf {t}_{N}}^{y},\kappa _{\mathbf {t}_{N}}^{y}\right \}$ of hyperparameters. The quantities $\nu _{\mathbf {t}_{N}}^{y}$ and $\kappa _{\mathbf {t}_{N}}^{y}$ define our certainty about the prior knowledge (here, the set of SDEs presenting the dynamics of the model). If we take the conjugate priors for mean and covariance when the sampling is Gaussian, i.e., a normal-inverse-Wishart distribution (which depends on **t**_*N*_), then

(25) $$ {\small{\begin{aligned} {}\pi& \left(\mathbf{\theta}_{\mathbf{t}_{N}}^{y}\right)\propto |\boldsymbol{\Sigma }_{\mathbf{t}_{N}}^{y}|^{-(\kappa_{\mathbf{t}_{N}}^{y}+Np+1)/2}\text{exp}\left(-\frac{1}{2}\text{tr}\left(\breve{\boldsymbol{\Psi }}_{\mathbf{t}_{N}}^{y}\left(\boldsymbol{\Sigma }_{\mathbf{t}_{N}}^{y}\right)^{-1}\right)\right) \\ & \times\! |\boldsymbol{\Sigma }_{\mathbf{t}_{N}}^{y}|^{-1/2}\text{exp}\!\left(\!\!-\frac{\nu_{\mathbf{t}_{N}}^{y}}{2}\!\left(\boldsymbol{\mu }_{\mathbf{t}_{N}}^{y}\,-\,\mathbf{m}_{\mathbf{t}_{N}}^{y}\right)^{T}\!\left(\boldsymbol{\Sigma }_{\mathbf{t}_{N}}^{y}\right)^{-1}\!\left(\boldsymbol{\mu}_{\mathbf{t}_{N}}^{y}\,-\,\mathbf{m}_{\mathbf{t}_{N}}^{y}\right)\!\right)\!, \end{aligned}}}  $$

with $\boldsymbol {\mu }_{\mathbf {t}_{N}}^{y}$ and $\boldsymbol {\Sigma }_{\mathbf {t}_{N}}^{y}$ defined in (6) and (7). Therefore, the above assumption (ii) on the nature of the prior information means that

(26) $$ \begin{aligned} &\breve{\mathbf{m}}_{\mathbf{t}_{N}}^{y}=\mathbf{m}_{\mathbf{t}_{N}}^{y} \\ &\breve{{\boldsymbol{\Psi }}}_{\mathbf{t}_{N}}^{y}=\left(\kappa_{\mathbf{t}_{N}}^{y}-Np-1\right){\boldsymbol{\Psi }}_{\mathbf{t}_{N}}^{y}, \end{aligned}   $$

with $\mathbf {m}_{\mathbf {t}_{N}}^{y}$ defined by (16) and (20) and ${\boldsymbol {\Psi }}_{\mathbf {t}_{N}}^{y}$ defined by (17) and (21). To see (26), note that from (25) and independence of $\boldsymbol {\mu }_{\mathbf {t}_{N}}^{y}$ and $ \boldsymbol {\Sigma }_{\mathbf {t}_{N}}^{y}$, we have $E_{\pi }\left [\!\boldsymbol {\mu }_{\mathbf {t}_{N}}^{y}\right ]=\breve {\mathbf {m}}_{\mathbf {t}_{N}}^{y}$ and $ E_{\pi }\left [\!\boldsymbol {\Sigma }_{\mathbf {t}_{N}}^{y}\right ]=\frac {\breve {{\boldsymbol {\Psi }}}_{\mathbf {t}_{N}}^{y}}{\kappa _{\mathbf {t}_{N}}^{y}-Np-1} $ (the latter is the mean of an inverse-Wishart distribution). The more confident we are about an a priori set of SDEs that is supposed to represent the underlying stochastic processes at **t**_*N*_^*y*^, the larger we might choose the values of $\nu _{\mathbf {t}_{N}}^{y}$ and $\kappa _{\mathbf {t}_{N}}^{y}$ and the more concentrated become the priors of the mean and covariance about $\mathbf {m}_{\mathbf {t}_{N}}^{y}$ and ${\boldsymbol {\Psi }}_{\mathbf {t}_{N}}^{y}$, respectively. To ensure a proper prior distribution, we assume $\breve {\boldsymbol {\Psi }}_{\mathbf {t}_{N}}^{y}$ is positive definite, $\kappa _{\mathbf {t}_{N}}^{y}>Np-1$, and $\nu _{\mathbf {t}_{N}}^{y}>0$ for all **t**_*N*_ (cf. p. 126 in [16], p. 178 in [17], and p. 427 in [3]).

Given the preceding framework for uncertainty in the BCGP model, the optimal Bayesian classification theory can be directly adapted. Specifically, the normal-inverse-Wishart distribution prior as defined in (25) and the independence of $\mathbf {X}_{\mathbf {t}_{N}}^{y}(\omega _{s})$ from training sample paths resemble the same set of conditions as in [6], i.e., having a normal-inverse-Wishart distribution prior and independence of future data points from training data points. As a result, we can follow the same set of machinery to find the *effective class-conditional distributions of the processes* (similar to equation (64) in [6]) and from there obtain the optimal discriminant. Therefore, extending the dimensionality of the problem to *Np* and using the set of parameters $\left \{\breve {\mathbf {m}}_{\mathbf {t}_{N}}^{y},\breve {\boldsymbol {\Psi }}_{\mathbf {t}_{N}}^{y},\nu _{\mathbf {t}_{N}}^{y},\kappa _{ \mathbf {t}_{N}}^{y}\right \}$ in the discriminant presented by Eq. (65) in [6] yields

(27) $$ \begin{aligned} {}\psi_{\mathbf{t}_{N}}^{\text{OBC}}\left(\mathbf{x}_{\mathbf{t}_{N}}^{y}(\omega_{s})\right)& =K\left(1+\frac{1}{k^{0}}\left(\mathbf{x}_{\mathbf{t}_{N}}^{y}(\omega_{s})-{\mathbf{m}}_{\mathbf{t}_{N}}^{0\;\ast }\right)^{T}{\boldsymbol{\Pi}}_{\mathbf{t}_{N}}^{0\;-1}\right.\\ &\quad\times\left.\left(\mathbf{x}_{\mathbf{t}_{N}}^{y}(\omega_{s})-{\mathbf{m}}_{\mathbf{t}_{N}}^{0\;\ast }\right)\vphantom{\frac{0}{0}}\right)^{k^{0}+Np} \\ &\quad-\left(1+\frac{1}{k^{1}}\left(\mathbf{x}_{\mathbf{t}_{N}}^{y}\left(\omega_{s}\right)-{\mathbf{m}}_{\mathbf{t}_{N}}^{1\;\ast}\right)^{T}{\boldsymbol{\Pi}}_{\mathbf{t}_{N}}^{1\;-1}\right.\\ &\quad\times\left.\left(\mathbf{x}_{\mathbf{t}_{N}}^{y}(\omega_{s})-{\mathbf{m}}_{\mathbf{t}_{N}}^{1\;\ast}\right)\vphantom{\frac{0}{0}}\right)^{k^{1}+Np}, \end{aligned}  $$

where

(28) $$ {{\begin{aligned} {}K\!=\left(\frac{\alpha_{1}}{1-\alpha_{0}}\right)^{2}\left(\frac{k^{0}}{k^{1}} \right)^{Np}\frac{|{\boldsymbol{\Pi }}_{\mathbf{t}_{N}}^{0}|}{|{\boldsymbol{\Pi}}_{\mathbf{t}_{N}}^{1}|}\left(\frac{\Gamma (k^{0}/2)\Gamma ((k^{1}+pN)/2)}{\Gamma (k^{1}/2)\Gamma ((k^{0}+pN)/2)}\right)^{2}, \end{aligned}}}  $$

with

(29) $$ {{\begin{aligned} &{\boldsymbol{\Pi}}_{\mathbf{t}_{N}}^{y}=\frac{\nu_{\mathbf{t}_{N}}^{y\;\ast}+1}{\left(\kappa_{\mathbf{t}_{N}}^{y\;\ast }-Np+1\right)\nu_{\mathbf{t}_{N}}^{y\;\ast }}{\boldsymbol{\Psi}}_{\mathbf{t}_{N}}^{y\;\ast },\\ &{\boldsymbol{\Psi}}_{\mathbf{t}_{N}}^{y\;\ast}\,=\,\breve{\boldsymbol{\!\Psi}}_{\mathbf{t}_{N}}^{y}\!\,+\,\!(n^{y}\!\,-\,\!1)\hat{\mathbf{\Sigma }}_{\mathbf{t}_{N}}^{y}\!\,+\,\!\frac{\nu_{\mathbf{t}_{N}}^{y}n^{y}}{\nu_{\mathbf{t}_{N}}^{y}+n^{y}}\!\left(\hat{\boldsymbol{\mu}}_{\mathbf{t}_{N}}^{y}\!\,-\,\breve{\mathbf{m}}_{\mathbf{t}_{N}}^{y}\right)\!\!\left(\hat{ \boldsymbol{\mu}}_{\mathbf{t}_{N}}^{y}\,-\,\breve{\mathbf{m}}_{\mathbf{t}_{N}}^{y}\right)^{T}\!, \\ &\nu_{\mathbf{t}_{N}}^{y\;\ast }=\nu_{\mathbf{t}_{N}}^{y}+n^{y},\quad \kappa_{\mathbf{t}_{N}}^{y\;\ast }=\kappa_{\mathbf{t}_{N}}^{y}+n^{y},\quad k^{y}=\kappa_{\mathbf{t}_{N}}^{y\;\ast }-Np+1, \\ &{\mathbf{m}}_{\mathbf{t}_{N}}^{y\;\ast }=\frac{\nu_{\mathbf{t}_{N}}^{y}\breve{\mathbf{m}}_{\mathbf{t}_{N}}^{y}+n^{y}\hat{ \boldsymbol{\mu}}_{\mathbf{t}_{N}}^{y}}{\nu_{\mathbf{t}_{N}}^{y}+n^{y}}, \end{aligned}}}   $$

where $\breve {\mathbf {m}}_{\mathbf {t}_{N}}^{y}$ and $\breve {{\boldsymbol { \Psi }}}_{\mathbf {t}_{N}}^{y}$ are determined from (26), and $\hat { \mathbf {\Sigma }}_{\mathbf {t}_{N}}^{y}$ and $\hat {\boldsymbol {\mu }}_{\mathbf {t}_{N}}^{y}$ are the sample mean and sample covariance matrix obtained by using the sample path training sets $\mathbf {S}_{\mathbf {t}_{N}}^{0}$ and $\mathbf {S}_{\mathbf {t}_{N}}^{1}$ as follows:

(30) $$ {{\begin{aligned} {} \hat{\boldsymbol{\mu}}_{\mathbf{t}_{N}}^{y}&=\frac{1}{n^{y}}\sum_{i=1}^{n^{y}} \mathbf{x}_{\mathbf{t}_{N}}^{y}(\omega_{i}) \,,\\ {}\hat{\boldsymbol{\Sigma}}_{\mathbf{t}_{N}}^{y}&=\frac{1}{n^{y}-1}\sum_{i=1}^{n^{y}}\left(\mathbf{x}_{\mathbf{t}_{N}}^{y}(\omega_{i})\,-\,\hat{\boldsymbol{\mu}}_{\mathbf{t}_{N}}^{y}\right)\left(\mathbf{x}_{ \mathbf{t}_{N}}^{y}(\omega_{i})\,-\,\hat{\boldsymbol{\mu}}_{\mathbf{t}_{N}}^{y}\right)^{T}\,. \end{aligned}}}  $$

As opposed to Section 4.1, where the discriminant can be applied to any future sample path with an arbitrary observation time vector, here, the discriminant depends on both the future and training observation time vectors. Thus, if the future observation time vector **t**_*L*_^*y*^ contains only a set of time points *t*_*i*_ where *t*_*i*_∈**t**_*N*_^*y*^, one may easily apply the optimal discriminant. This is easily doable by reducing the dimensionality of the problem by considering the training sample paths only at **t**_*L*_^*y*^, i.e., by discarding the training sample points at those **t**_*N*_^*y*^ not in **t**_*L*_^*y*^(denoted by **t**_*N*_^*y*^∖**t**_*L*_^*y*^). However, solving the case where **t**_*L*_^*y*^ includes time points not included in **t**_*N*_^*y*^ is more difficult and requires further study. In this case, although one is able to construct the class of prior knowledge for **t**_*L*_^*y*^ (i.e., constructing ${\boldsymbol {\mu }} _{\mathbf {t}_{N}}^{y}$ and $\boldsymbol {\Psi }_{\mathbf {t}_{N}}^{y}$), the paucity of training sample paths at **t**_*L*_^*y*^∖**t**_*N*_^*y*^ does not permit employing (27).

## Performance analysis

In this section, we analyze the effect of prior knowledge in the form of stochastic differential equations on the performance of the stochastic discriminant, $\psi _{\mathbf {t}_{N}}^{OBC}\left (\mathbf {x}_{\mathbf {t} _{N}}^{y}(\omega _{s})\right)$, defined by (27)–(29). As the metric of performance, we take the true error averaged over the sampling space. The true error of a discriminant trained on an observation time vector **t**_*N*_, i.e., $\psi _{\mathbf {t}_{N}\phantom {\dot {i}\!}}(.)$, is the probability of misclassification, which by considering (11) is defined as

(31) $$ {{\begin{aligned} {}\epsilon_{\mathbf{t}_{N}}\!\,=\,\sum_{y=0}^{1}\alpha_{\mathbf{t}_{N}}^{y}P\!\left((-1)^{y}\psi_{\mathbf{t}_{N}}\!\left(\mathbf{X}_{\mathbf{t}_{N}}^{y}(\omega_{s})\right)\!>\!0\!\mid\!\mathbf{S}_{\mathbf{t}_{N}}^{0},\mathbf{S}_{\mathbf{t}_{N}}^{1}, \mathbf{X}_{\mathbf{t}_{N}}^{y}(\omega_{s})\!\in\! \mathbf{X}^{y}\right)\!, \end{aligned}}}  $$

where **X** ^*y*^ denotes the class-conditional process that generates the future sample path $\mathbf {X}_{\mathbf {t}_{N}}^{y}(\omega _{s})$ (we assume independence of future sample paths from training sample paths), $\mathbf {S}_{\mathbf {t}_{N}}^{y}$ denotes the set of training sample paths from class *y*, and $\alpha _{\mathbf {t}_{N}}^{y}$ is the mixing probability of the class-conditional process.

Recall that in this work, we consider a separate sampling scheme. With separate sampling in a classical binary classification problem where sample points are generated from two class-conditional densities, there is no sensible estimate of prior probabilities of classes from the sample [15]. In that case, either the ratio of the number of sample points in either class to the total sample size needs to reflect the corresponding prior probability of the class or the prior probabilities need to be known a priori; otherwise, classification rules or error estimation rules suffer performance degradation [15, 18, 19]. The same argument applies to this work in which we consider a binary classification of sample paths that are generated from two class-conditional processes under a separate sampling scheme. In this regard, we assume that the prior probability $\alpha _{\mathbf {t}_{N}}^{y}$ is known a priori.

Taking expectation over the sample space, that is over the mixture of Gaussian processes with the means and covariance matrices defined by (16), (20), (17), and (21), yields

(32) $$ {\small{\begin{aligned} {}E[\!\epsilon_{\mathbf{t}_{N}}]\,=\,\sum_{y=0}^{1}\alpha_{\mathbf{t}_{N}}^{y}P\!\left((-1)^{y}\psi_{\mathbf{t}_{N}}\!\left(\mathbf{X}_{\mathbf{t}_{N}}^{y}(\omega_{s})\right)\!>\!0\!\mid\!\! \,\mathbf{X}_{\mathbf{t}_{N}}^{y}(\omega_{s})\in\! \mathbf{X}^{y}\right). \end{aligned}}}   $$

As benchmarks for evaluating the performance of $\psi _{\mathbf {t}_{N}}^{\text {OBC}} \left (\mathbf {x}_{\mathbf {t}_{N}}^{y}(\omega _{s})\right)$, we compare its performance to (1) the performance of the stochastic QDA, $\psi _{\mathbf {t}_{N}}^{\text {QDA}}\left (\mathbf {X}_{\mathbf {t}_{N}}^{y}(\omega _{s})\right)$, which is defined by (23) and (24), where *l*_*y*_=*n*_*y*_, with *n*_*y*_ indicating the number of available sample paths, and (2) the performance of a Bayes classifier obtained by plugging (16), (17), (20), and (21), into (24).

### Synthetic experiments

#### Experimental set-up

The following steps are used to set up the experiments:

1. To fix the ground-truth model governing the underlying dynamics of the data, we consider a set of three-dimensional SDEs (*p*=3) defined by (15) along with the following set of parameters:
  (33) $$ {\small{\begin{aligned} \mathbf{A}^{0}(t)&=\mathbf{A}^{1}(t)=[\!0.01,0.01,0.01]^{T},\\ \mathbf{a}^{0}(t)&=\mathbf{a}^{1}(t)=[\!0,0,0]^{T}, \\ &\mathbf{X}_{t_{0}}^{0}(\omega)=[\!0,0,0]^{T},\quad \mathbf{X}_{t_{0}}^{1}(\omega)=[\!0.25,0.25,0.25]^{T}, \\ &\mathbf{B}^{0}(t)=\mathbf{B}^{1}(t)=0.1\!\times \left\{ \begin{array}{ll} \sigma^{2}=1 &\text{diagonal elements} \\ \rho =0.4 & \text{otherwise} \end{array}\right.. \end{aligned}}}   $$
  The only difference between the SDEs describing **X** ^0^ and **X** ^1^ is in the constant initial conditions. Figure 1 presents a single sample path of these two three-dimensional processes for 0≤*t*≤100.

**Fig. 1:**
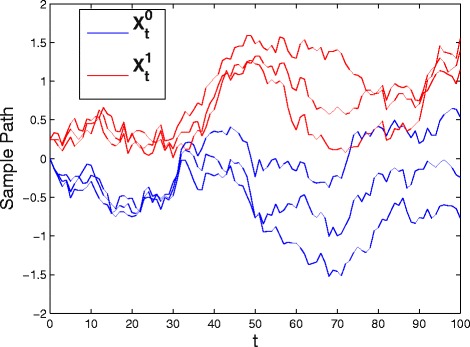
A single sample path taken from the two three-dimensional processes described by the set of parameters introduced in (33)
2. Use the ground-truth set of SDEs to generate a set of training sample paths, $\mathbf {S}_{\mathbf {t}_{N}}^{y}$, of size *n* ^*y*^ for class *y*=0,1. We let *n* ^0^=*n* ^1^=*n*, where *n*∈, let the length of the observation time vector be *N*=20, and take [ *t*_1_,*t*_2_,...,*t*_*N*_] such that *t*_*i*_−*t*_*i*−1_=1, *i*=2,…,20.
3. Use the ground-truth set of SDEs to generate a set of test sample paths, $\mathbf {S}_{\mathbf {t}_{N}}^{y,\,\text {test}}$, of size *n* ^*y*, test^=2,000 for class *y*=0,1, where *n* ^0, test^=*n* ^1, test^=*n* ^test^.
4. Use $\mathbf {S}_{\mathbf {t}_{N}}^{0}\cup \mathbf {S}_{\mathbf {t} _{N}}^{1}$ to train the stochastic QDA, $\psi _{\mathbf {t}_{N}}^{\text {QDA}}\left (\mathbf {x}_{\mathbf {t}_{N}}^{y}(\omega _{s})\right)$, which is defined by (23) and (24) with *l* ^*y*^=*n* ^*y*^. Apply the trained classifier to the set of test sample paths, $\mathbf {S}_{\mathbf {t} _{N}}^{0,\,\text {test}}\cup \mathbf {S}_{\mathbf {t}_{N}}^{1,\,\text {test}}$, to determine the true error, $\epsilon _{\mathbf {t}_{N}}^{\text {QDA}}$, which is defined by replacing $\psi _{\mathbf {t}_{N}}\left (\mathbf {X}_{\mathbf {t}_{N}}^{y}(\omega _{s})\right)$ with $\psi _{\mathbf {t}_{N}}^{\text {QDA}}\left (\mathbf {X}_{\mathbf {t}_{N}}^{y}(\omega _{s})\right)$ in (31). This procedure obtains an accurate estimate of true error.
5. Assume a set of SDEs obtained from prior knowledge (a priori SDEs). Let this a priori set of SDEs be presented by replacing **A** ^*y*^(*t*), **B** ^*y*^(*t*), **A** ^*y*^(*t*), and $\mathbf {X} _{t_{0}}^{y}(\omega)$ in (15) with $\tilde {\mathbf {A}}^{y}(t)$, $\tilde {\mathbf {B}}^{y}(t)$, $\tilde {\mathbf {A}}^{y}(t)$, and $\tilde {\mathbf { X}}_{t_{0}}^{y}(\omega)$, respectively. To examine the effects of deviations in the drift vector and dispersion matrix in the a priori set of SDEs from the ground-truth model introduced in (33), we assume
$\tilde {\mathbf {A}}^{0}(t)={\mathbf {A}}^{0}(t)$, $\tilde {\mathbf {B}} ^{0}(t)={\mathbf {B}}^{0}(t)$, $\tilde {\mathbf {X}}^{0}_{t_{0}}(\omega)= \mathbf {X}_{t_{0}}^{0}(\omega)$, $\tilde {\mathbf {X}}^{1}_{t_{0}}(\omega)= \mathbf {X}_{t_{0}}^{1}(\omega)$, $\tilde {\mathbf {a}}^{0}(t)=\mathbf {a} ^{0}(t),\tilde {\mathbf {a}}^{1}(t)=\mathbf {a}^{1}(t)$.
  To study the effect of shift in the drift vector, we take $\tilde { \mathbf {A}}^{1}(t)={\mathbf {A}}^{1}(t)+[\!\Delta \mu,\Delta \mu,\Delta \mu ]^{T}$, where *Δ* *μ*=0,0.1,0.2,0.3. Here we assume $\tilde {\mathbf {B}} ^{1}(t)={\mathbf {B}}^{1}(t)$.
  To study the effect of shift in the dispersion matrix, we assume the off-diagonal elements of $\tilde {\mathbf {B}}^{1}(t)$ are defined by replacing *ρ* with *ρ*_*d*_ in (33), where *ρ*_*d*_−*ρ*=*Δ* *ρ*=0,0.03,0.06,0.1. Here we assume $\tilde {\mathbf {A}} ^{1}(t)={\mathbf {A}}^{1}(t)$.
  The hyperparameters defining our uncertainty about the specific choice of a priori SDEs (in fact, about the resultant prior distributions) are $\nu _{\mathbf {t}_{N}}^{0}=\nu _{\mathbf {t}_{N}}^{1}=\kappa _{\mathbf {t} _{N}}^{0}=\kappa _{\mathbf {t}_{N}}^{1}=Np+\kappa $. The choice of *N* *p*+*κ*, *κ*=20,50,100,500, is made to have proper prior distributions (see Section 4.2).
6. Generate 2,000 sample paths from the a priori set of SDEs introduced in Step 5. These sample paths are used to calculate the hyperparameters $\mathbf {m}_{\mathbf {t}_{N}}^{y}$ and ${\boldsymbol {\Psi }}_{ \mathbf {t}_{N}}^{y}$ being used in (26) (alternatively, one may solve (16), (17), (20), and (21) directly and use them in (26)).
7. Use $\mathbf {m}_{\mathbf {t}_{N}}^{y}$ and ${\boldsymbol {\Psi }}_{ \mathbf {t}_{N}}^{y}$ obtained from Step 6 along with $\mathbf {S}_{\mathbf {t} _{N}}^{0}\cup \mathbf {S}_{\mathbf {t}_{N}}^{1}$ to train $\psi _{\mathbf {t} _{N}}^{\text {OBC}}\left (\mathbf {x}_{\mathbf {t}_{N}}^{y}(\omega _{s})\right)$, which is defined in (27). Apply the trained classifier to the set of test sample paths, $\mathbf {S}_{\mathbf {t}_{N}}^{0,\,\text {test}}\cup \mathbf {S}_{ \mathbf {t}_{N}}^{1,\,\text {test}}$, to determine the true error, $\epsilon _{ \mathbf {t}_{N}}^{\text {OBC}}$, which is defined by replacing $\psi _{\mathbf {t}_{N}} \left (\mathbf {X}_{\mathbf {t}_{N}}^{y}(\omega _{s})\right)$ with $\psi _{\mathbf {t}_{N}}^{\text {OBC}}\left (\mathbf {x}_{\mathbf {t}_{N}}^{y}(\omega _{s})\right)$ in (31).
8. Repeat Steps 2 through 7 a total of *T*=1,000 times to estimate $ E\left [\epsilon _{\mathbf {t}_{N}}^{\text {QDA}}\right ]$ and $E\left [\epsilon _{\mathbf {t}_{N}}^{\text {OBC}}\right ] $.
9. Generate 2,000 sample paths from the ground-truth set of SDEs introduced in (33). Use these sample paths to train the stochastic QDA, $\psi _{\mathbf {t}_{N}}^{\text {QDA}}\left (\mathbf {x}_{\mathbf {t} _{N}}^{y}(\omega _{s})\right)$, which is defined by (23) and (24) with *l* ^*y*^=2,000. This provides an accurate estimate of the Bayes (optimal) classifier. Apply this classifier to $\mathbf {S}_{\mathbf {t} _{N}}^{0,\,\text {test}}\cup \mathbf {S}_{\mathbf {t}_{N}}^{1,\,\text {test}}$ to obtain the Bayes error, which is a lower bound on the error of any classifier. Note that in our experiments obtaining the Bayes error is possible since we have complete knowledge of the underlying ground-truth models.

#### Results

**Fig. 2:**
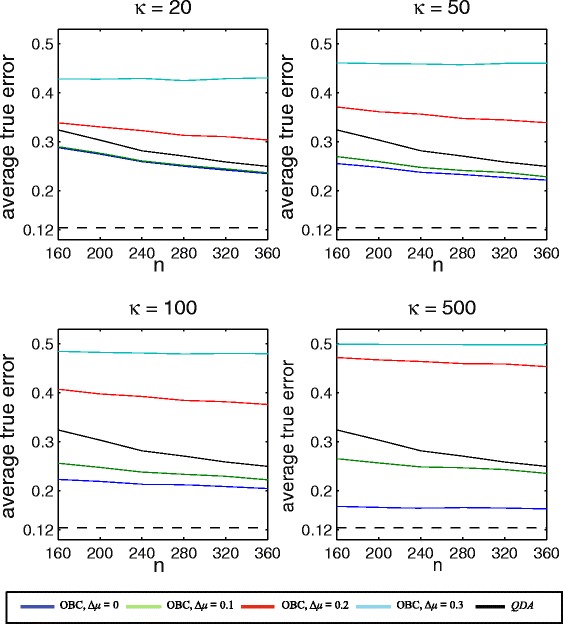
Expected true error, $E\left [\protect \epsilon ^{\text {QDA}}_{\mathbf {t}_{N}}\right ]$ and $E\left [\protect \epsilon ^{\text {OBC}}_{\mathbf {t}_{N}}\right ]$, as a function of number of training sample paths in each class and various choices of *Δ* *μ* and *κ*. The *dashed line* shows the Bayes error

Figure 2 shows the effect of a shift in the drift vector from the ground-truth model via plots of the expected true error of $\psi _{\mathbf {t}_{N}}^{\text {QDA}}(.)$ and $\psi _{\mathbf {t}_{N}}^{\text {OBC}}(.)$ as functions of the size of training sample paths and *κ* for *y*=0,1, $\tilde {\mathbf {B}}^{y}(t)={\mathbf {B}}^{y}(t)$, $\tilde {\mathbf {X}} ^{y}_{t_{0}}(\omega)=\mathbf {X}_{t_{0}}^{y}(\omega)$, $\tilde {\mathbf {A}} ^{0}(t)={\mathbf {A}}^{0}(t)$, and $\tilde {\mathbf {A}}^{1}(t)={\mathbf {A}} ^{1}(t)+[\!\Delta \mu,\Delta \mu,\Delta \mu ]^{T}$, where *Δ* *μ*=0,0.1,0.2,0.3. If the set of a priori SDEs is equivalent or close to the ground-truth model, e.g., *Δ* *μ*=0 or *Δ* *μ*=0.1, then $\psi _{\mathbf {t}_{N}}^{\text {OBC}}(.)$ outperforms $\psi _{\mathbf {t}_{N}}^{\text {QDA}}(.)$ for a wide range of training sample sizes and *κ*. The more the prior distribution generated from the set of a priori SDEs is concentrated about the true underlying parameters of the model and the larger *κ*, the better is the performance achieved by using $\psi _{\mathbf {t}_{N}}^{\text {OBC}}(.)$.

**Fig. 3:**
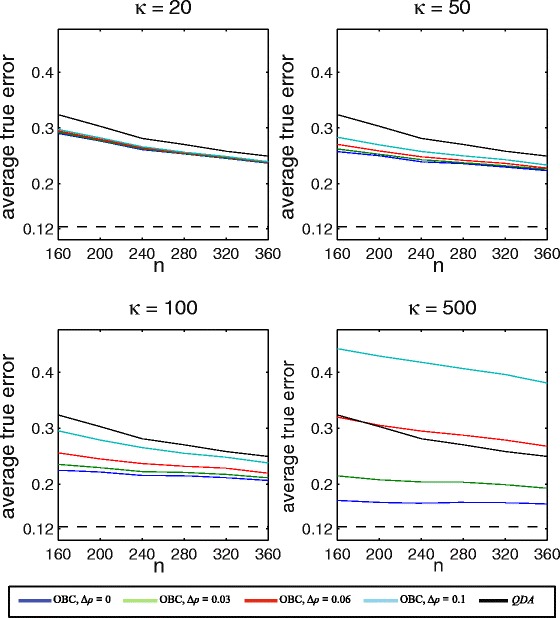
Expected true error, $E\left [\epsilon ^{\text {QDA}}_{\mathbf {t}_{N}}\right ]$ and $E\left [\epsilon ^{\text {OBC}}_{\mathbf {t}_{N}}\right ]$, as a function of number of training sample paths in each class and various choices of *Δ* *ρ* and *κ*. The *dashed line* shows the Bayes error

Figure 3 presents the effect of the discrepancy between the dispersion matrix of the ground-truth model and that of the a priori set of SDEs. Again, the closer the prior knowledge is to the ground-truth model and the larger *κ*, the better is the performance achieved by using $\psi _{\mathbf {t}_{N}}^{\text {OBC}}(.)$.

### An experiment inspired by a model of the evolutionary process

In this section, we use a form of an Ornstein-Uhlenbeck process introduced in [20] for modeling the evolutionary change of species. This model has been recently employed by [21] to simulate quantitative trait data as a function of single nucleotide polymorphism (SNP) states. The model is presented by the following SDE:

(34) $$ \mathrm{d}{X}_{t}^{y}=-\beta^{y}\left[{\!X_{t}^{y}}-\theta^{y}\right]\,\mathrm{d}t+\sigma^{y} \mathrm{d}{W}_{t}^{y},\quad {X_{0}^{y}}={X_{a}^{y}},   $$

where ${X}_{t}^{y}$ is the quantitative trait value in a species *y*, *θ* ^*y*^ is the primary target value of the trait, ${X_{a}^{y}}$ is the mean state in an ancestor *a*, and ${W}_{t}^{y}$ represents Brownian motion. The parameter *β* ^*y*^ is the rate of adaptation of species *y* to the target value—a low rate of adaptation means very slow evolution while a large *β* ^*y*^ practically indicates an instantaneous adaptation. The parameter *σ* ^*y*^ is an indicator of perturbation due to random selective factors such as random mutations and environmental fluctuations [20]. Similar to [21], we assume the value of the primary target is constant over the history of the species. Nevertheless, the model in (34) can be extended to include situations where the primary target can change over the evolutionary history of the species (see [20]).

**Fig. 4:**
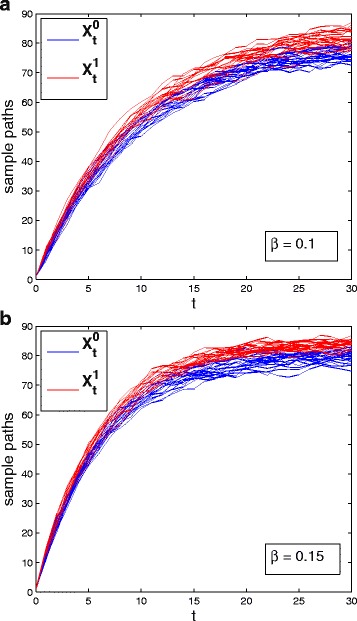
Multiple sample paths taken from the two one-dimensional evolutionary processes for two values of adaptation rate, (**a**): *β*=0.1; (**b**): *β*=0.15

Using the model of (34), we generate the evolutionary histories of a quantitative trait of two species, 0 and 1, over a time span of 30 million years with time steps of 1 million years. Similarly to [20, 21], to fix the ground-truth model that generates the data, we vary values of *β* ^*y*^, take *σ* ^*y*^=1, and assume *θ* ^0^=80 and *θ* ^1^=85. Furthermore, we assume both species have a common ancestor at the state ${X_{a}^{y}}=1$. Figure 4 presents 20 sample paths from each of these evolutionary processes for the case where *β* ^0^=*β* ^1^=*β*, *β*=0.1 (Fig. 4 a) and *β*=0.15 (Fig. 4 b). A larger *β* indicates a faster adaptation of species to the target value. The problem considered here is to use a set of a priori SDEs in constructing a classifier to differentiate the evolutionary history of an *n*-size population of species 0 from an *n*-size population of species 1, where *n*∈[ 60,140].

The general protocol for evaluating the performance of *ψ* **t**_*N*_OBC(.) is similar to Section 5.1, except for replacing the ground-truth model (33) with (34) and using the following the step instead of Step 5:

- Assume a set of SDEs obtained from prior knowledge (a priori SDEs). Let this a priori set of SDEs be presented by replacing *β* ^*y*^, *θ* ^*y*^, ${X_{a}^{y}}$, and *σ* ^*y*^ by $\tilde {\beta } ^{y}$, $\tilde {\theta }^{y}$, $\tilde {X}_{a}^{y}$, and $\tilde {\sigma }^{y}$, respectively, in (34). To examine the effect of deviation of the adaptation rate in the a priori set of SDEs from the ground-truth model, we let $ \tilde {\theta }^{y}={\theta }^{y}$, $\tilde {\sigma }^{y}={\sigma }^{y}$, $ \tilde {X}_{a}^{y}={X}^{y}$, and $\tilde {\beta }^{0}={\beta }^{0}$ and take $ \tilde {\beta }^{1}={\beta }^{1}+\Delta \beta $.

#### Results

**Fig. 5:**
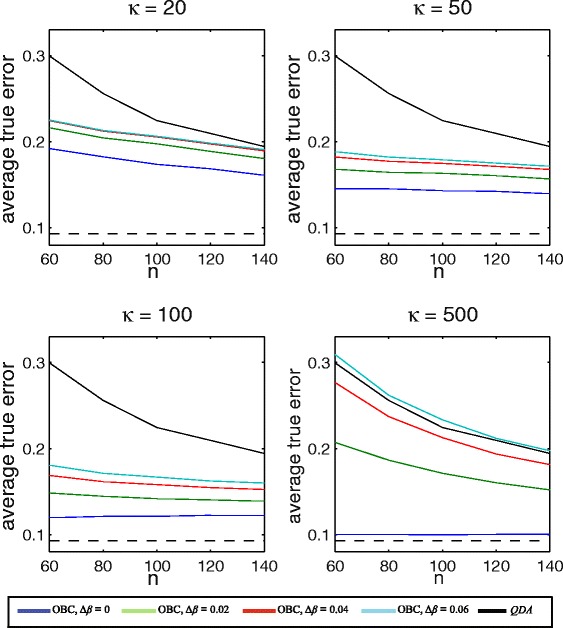
Expected true error, $E\big [\protect \epsilon ^{\text {QDA}}_{\mathbf {t}_{N}}\big ]$ and $E\left [\protect \epsilon ^{\text {OBC}}_{\mathbf {t}_{N}}\right ]$, as a function of number of training sample paths in each class and various choices of *Δ* *β* and *κ* and for *β*=0.1. The *dashed line* shows the Bayes error

**Fig. 6:**
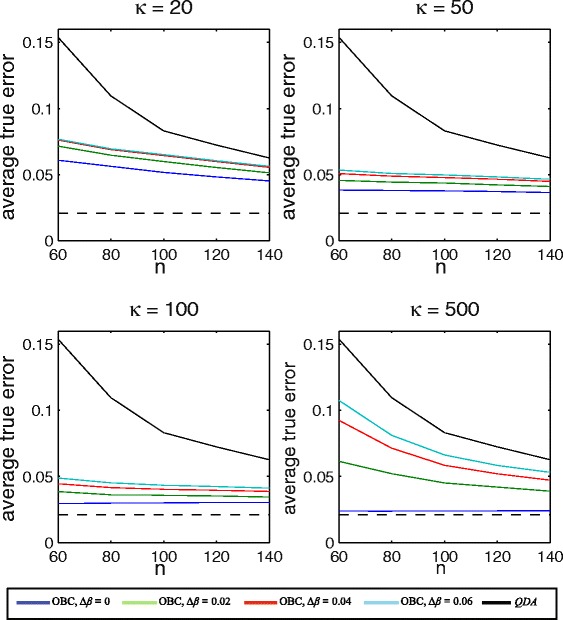
Expected true error, $E\big [\protect \epsilon _{\mathbf {t}_{N}}^{\text {QDA}}\big ]$ and $E\left [\protect \epsilon _{\mathbf {t}_{N}}^{\text {OBC}}\right ]$, as a function of number of training sample paths in each class and various choices of *Δ* *β* and *κ* and for *β* =0.15. The *dashed line* shows the Bayes error

Figures 5 and 6 (*β*=0.1 and *β*=0.15, respectively) show the effect of a deviation from the true rate of adaptation to the target value by considering $\tilde {\beta }^{1}={\beta } ^{1}+\Delta \beta,$ where *Δ* *β*=0, 0.02, 0.04, 0.06. They provide plots of the expected true error of $\psi _{\mathbf {t}_{N}}^{\text {QDA}} (.)$ and $\psi _{\mathbf {t}_{N}}^{\text {OBC}}(.)$ as functions of the size of training sample paths and *κ*. In both figures, the closer the prior knowledge is to the ground-truth evolutionary models, the better is the performance achieved by using $\psi _{\mathbf {t}_{N}}^{\text {OBC}}(.)$. The performance deteriorates and eventually becomes worse than $\psi _{ \mathbf {t}_{N}}^{\text {QDA}}(.)$ as the prior knowledge diverges from the ground-truth model and the certainty about the prior knowledge increases (a bad combination when utilizing prior knowledge). In addition, comparing Figs. 5 and 6 shows that the smaller is the true value of *β* and the more destructive is a fixed deviation of prior knowledge from the true *β*.

## Conclusions

This paper provides the first instance in which prior knowledge in the form of SDEs is used to construct a prior distribution over an uncertainty class of feature-label distributions for the purpose of optimal classification. Given the ubiquity of small samples in biomedicine and other areas where sample data is expensive, time-consuming, limited by regulation, or simply unavailable, we have previously made the point that prior knowledge is the only avenue available. To achieve the mapping of SDE prior knowledge into a prior distribution, we have taken advantage of the form and Gaussianity of (12). This mapping is heavily dependent on the form of the SDEs, and one can expect widely varying mappings for different SDE settings.

In general, all parameters used in the a priori set of SDEs can affect the performance of $\psi _{\mathbf {t}_{N}}^{\text {OBC}}(.)$. These parameters include every element of the matrices $\tilde {\mathbf {A}}^{y}(t)$ and $\tilde {\mathbf {B}}^{y}(t)$ and all the elements of the vectors $\tilde { \mathbf {a}}^{y}(t)$ and $\tilde {\mathbf {X}^{y}}_{t_{0}}(\omega)$ used in the SDE’s presentation in (15). For example, in the experiment of the evolutionary change of species considered in (34), a deviation from each of the parameters, namely $\tilde {\beta }^{y}$, $\tilde {\sigma } ^{y} $, $\tilde {\theta }^{y}$, and $\tilde {X}_{a}^{y},$ can affect the performance of $\psi _{\mathbf {t}_{N}}^{\text {OBC}}(.)$. Although simulation studies can elucidate the effects of deviation of prior knowledge from the ground-truth model (as done herein), it would be beneficial to analytically characterize the performance of $\psi _{\mathbf {t}_{N}}^{\text {OBC}} (.)$ in terms of all the hyperparameters; however, this may be very difficult to accomplish. One possible approach may be to use an asymptotic Bayesian framework [22] to characterize the performance of $\psi _{\mathbf {t}_{N}}^{\text {OBC}}(.)$ in terms of sample size, dimensionality, and hyperparameters.

Recognizing that the construction of robust classifiers is simply a special case of optimal Bayesian classification where there are no sample data, so that the “posterior” is identical to the prior [7], the application of SDEs in this paper is at once applicable to optimal robust classification in a stochastic setting. Beyond that, one can consider the more general setting of optimal Bayesian robust filtering of random processes, where optimization across an uncertainty class of random processes, ideal and observed, is relative to process characteristics such as the auto- and cross-correlation functions [23]. Whereas in this paper we have considered using SDE prior knowledge to construct prior distributions governing uncertainty classes of feature-label distributions, it seems feasible to use SDE knowledge to construct prior distributions governing uncertainty classes of random-process characteristics in the case of optimal filtering. Of course, one must confront the increased abstraction presented by canonical representation of random processes [24, 25]; nevertheless, so long as one remains in the framework of second-order canonical expansions, it should be doable.

## Appendix

### Definition of ***q***-dimensional Wiener process

A one-dimensional Wiener process over [ 0,*T*] is a Gaussian process *W*={*W*_*t*_:*t*≥0} satisfying the following properties:

- For 0≤*t*_1_<*t*_2_<*T*, $W_{t_{2}}-W_{t_{1}}$ is distributed as $ \sqrt {t_{2}-t_{1}}N\left (0,\sigma ^{2}\right)$, where *σ*>0 (for the standard Wiener process, *σ*=1).
- For 0≤*t*_1_<*t*_2_<*t*_3_<*t*_4_<*T*, $W_{t_{4}}-W_{t_{3}}$ is independent of $W_{t_{2}}-W_{t_{1}}$.
- *W*_0_=0 with probability 1.
- The sample paths of *W* are almost surely continuous everywhere.

In general, a *q*-dimensional Wiener process is defined using the homogenous Markov process **X**_*t*_ for *t*∈[*t*_0_,*T*]. Let $P(t_{1},x;t_{2}, B)=P(\mathbf {X}_{t_{2}\phantom {\dot {i}\!}} \in B|\mathbf {X}_{t_{1}\phantom {\dot {i}\!}}=x)$ denote the transition probabilities of a Markov process **X**_*t*_ for *t*_1_<*t*_2_. For fixed values of *t*_1_, *x*, and *t*_2_, *P*(*t*_1_,*x*;*t*_2_,.) is a probability function (measure) on the *σ*-algebra $\mathcal {B}$ of Borel subsets of the sample space *R* ^*q*^. Intuitively, *P*(*t*_1_,*x*;*t*_2_,*B*) is the probability that the process be in the set $B\in \mathcal {B}$ at time *t*_2_ given it was in state *x* at time *t*_1_. A Markov process is homogenous with respect to *t* if its transition probability *P*(*t*_1_,*x*;*t*_2_,*B*) is stationary. That is, for *t*_0_<*t*_1_<*t*_2_<*T* and *t*_0_<*t*_1_+*u*<*t*_2_+*u*<*T*, it satisfies

(35) $$ P(t_{1}+u,x;t_{2}+u, B)=P(t_{1},x;t_{2}, B).  $$

In this case *P*(*t*_1_,*x*;*t*_2_,*B*) is commonly denoted by *P*(*t*_2_−*t*_1_,*x*;*B*). A *q*-dimensional Wiener process is a *q*-dimensional homogenous Markov process defined on [0,*∞*) with stationary transition probability defined by a multivariate Gaussian distribution as follows:

(36) $$ P(t,x;B)=\int_{B} \frac{1}{(2\pi t)^{d/2}}e^{-\frac{|y-x|^{2}}{2t}} dy.  $$

Therefore, each dimension of a *q*-dimensional Wiener process is a one-dimensional Wiener process per se.

### Computational complexity

The computational complexity of the algorithm is determined by the computational cost of solving the set of SDEs from the Euler-Maruyama scheme (see Section 4.1) along with the computational cost of evaluating (27). The computational cost of the Euler-Maruyama scheme per sample path is inversely proportional to *Δ* *t* [26], where *Δ* *t*=*T*/*N*, with *T* and *N* being defined in Section 3. Thus, for *l*=*l* ^0^+*l* ^1^ sample paths, it is *O*(*l*/*Δ* *t*). In (27), the computational cost of evaluating $\mathbf {x}_{\mathbf {t}_{N}}^{y}(\omega _{s})-{\mathbf {m}}_{\mathbf {t}_{N}}^{0\;\ast },$ with *y*=0,1, breaks down to a computation of $\breve {\mathbf {m}}_{\mathbf {t}_{N}}^{y}$ and $\hat {\boldsymbol {\mu }}_{\mathbf {t}_{N}}^{y}$, which are operations with computational costs of *O*(*l* ^*y*^ *N* *p*) and *O*(*n* ^*y*^ *N* *p*), respectively.

Computation of ${{\boldsymbol {\Pi }}_{\mathbf {t}_{N}}^{y\;-1}}$ in (27) by Gaussian elimination is an *O*(max{*n* ^*y*^,*N* *p*}*N* ^2^ *p* ^2^)+*O*(*l* ^*y*^ *N* ^2^ *p* ^2^) operation (cf. section 3.7.2 in [27]). This will be further simplified because, in order to have a positive definite $\breve {{ \boldsymbol {\Psi }}}_{\mathbf {t}_{N}}^{y}$, we assume we generate many sample paths by solving the set of SDEs such that *l* ^*y*^>>*N* *p* (see Section 4.1), but since ${\boldsymbol {\Psi }}_{\mathbf {t}_{N}}^{y\;\ast }$ and ${{ \boldsymbol {\Pi }}_{\mathbf {t}_{N}}^{y\;-1}}$ defined in (29) become positive definite, we do not need to impose the condition of *n* ^*y*^>*N* *p*. Having a realistic assumption on the number of available sample paths, we can assume *l* ^*y*^>>*n* ^*y*^, and therefore, the computation of ${{\boldsymbol {\Pi } }_{\mathbf {t}_{N}}^{y\;-1}}$ becomes an *O*(*l* ^*y*^ *N* ^2^ *p* ^2^) calculation. Furthermore, the product of $\mathbf {x}_{\mathbf {t}_{N}}^{y}(\omega _{s})-{ \mathbf {m}}_{\mathbf {t}_{N}}^{0\;\ast }$ with ${{\boldsymbol {\Pi }}_{\mathbf { t}_{N}}^{y\;-1}}$ is an *O*(*N* ^2^ *p* ^2^) calculation. Altogether, by assuming 1/*Δ* *t*<(*N* *p*)^2^ and *k* ^0^+*k* ^1^+*N* *p*<(*N* *p*)^2^, the overall computational cost of $\psi _{\mathbf {t}_{N}}^{\text {OBC}}\left (\mathbf {x}_{\mathbf {t}_{N}}^{y}(\omega _{s})\right)$ is *O*(max{*l* ^0^,*l* ^1^}*N* ^2^ *p* ^2^).

Using a similar approach, we see that the computational cost of QDA, which is solely constructed by using *n* ^0^+*n* ^1^ training sample paths from classes 0 and 1 (i.e., no prior knowledge) is *O*(max{*n* ^0^,*n* ^1^}*N* ^2^ *p* ^2^). We also note that for computing QDA we need to have min{*n* ^0^,*n* ^1^}>*N* *p* because, otherwise, the sample covariance matrices used in QDA are not invertible.

## Additional file

Additional file 1
**Supplementary information.** I. Definition of QDA in a classical setting. II. Error estimation accuracy. III. Bayesian MMSE error estimator. IV. Review of literature pertaining to classification of stochastic processes.
