# Supplementary material for: Incorporating prior knowledge induced from stochastic differential equations in the classification of stochastic observations
Source: EURASIP J Bioinform Syst Biol. 2016 Jan 20;2016:2. doi: 10.1186/s13637-016-0036-y (PMC4720709; doi:10.1186/s13637-016-0036-y)
Supplement: Additional file 1 — Supplementary information. I. Definition of QDA in a classical setting. II. Error estimation accuracy. III. Bayesian MMSE error estimator. IV. Review of literature pertaining to classification of stochastic processes. [file 13637_2016_36_MOESM1_ESM.pdf]

Incorporating Prior Knowledge Induced from Stochastic Differential Equations in the Classification of Stochastic Observations Supplementary Information

Amin Zollanvari and Edward R. Dougherty

I. Definition of QDA in a Classical Setting

In order to make the manuscript self-contained, here we present the definition of Quadratic Discriminant Analysis (QDA) in a classical setting in which both class-conditional densities are Gaussian, class k having mean vector $\boldsymbol{\mu}_k \in \mathbb{R}^p$ and covariance matrix $\boldsymbol{\Sigma}_k \in \mathbb{R}^{p\times p}$ for $k = 0, 1$. In this case it is well-known that the Bayes classifier is given by $\psi(\mathbf{x}) = 1$ if $d_1(\mathbf{x}) \geq d_0(\mathbf{x})$, where $\mathbf{x}$ is a p-dimensional sample point, and the discriminant d_k is defined by

$$d_k(\mathbf{x}) = -\frac{1}{2}(\mathbf{x} - \boldsymbol{\mu}_k)^T \boldsymbol{\Sigma}_k (\mathbf{x} - \boldsymbol{\mu}_k) - \frac{1}{2}\ln 2\pi - \frac{p}{2}\ln\det[\boldsymbol{\Sigma}_k] \quad (1)$$

for $k = 0, 1$. The form of this equation shows that the decision boundary $d_1(\mathbf{x}) = d_0(\mathbf{x})$ is quadratic. In practice, when the means and covariance matrices are not known, they are replaced by the sample means and sample covariance matrices, and the resulting classification method is known as *quadratic discriminant analysis* (QDA).

In the special case where the covariance matrices are identical, then $\ln(\det[\boldsymbol{\Sigma}_k])$ can be dropped and the discriminant takes the form

$$d_k(\mathbf{x}) = -\frac{1}{2}(\mathbf{x} - \boldsymbol{\mu}_k)^T \boldsymbol{\Sigma}_k (\mathbf{x} - \boldsymbol{\mu}_k) \quad (2)$$

which is a linear function of $\mathbf{x}$ and produces hyperplane decision boundaries. When the classifier is designed from sample data, the means are replaced by the sample means, the covariance matrix is replaced

by the pooled sample covariance matrix, and the classification method is known as *linear discriminant analysis* (LDA).

QDA and LDA are derived under the Gaussian assumption but in practice can perform well so long as the class conditional densities are not too far from Gaussian and there is sufficient data to obtain good estimates of the relevant covariance matrices, the point being that the QDA and LDA classification rules involve finding the sample covariance matrices and sample means. Owing to the greater number of parameters to be estimated for QDA as opposed to LDA, one can proceed with smaller samples with LDA than with QDA.

II. Error Estimation Accuracy

Given a feature-label distribution, error estimation accuracy is commonly measured by the *mean-square error* (*MSE*), defined by $\mathrm{MSE}(\hat{\varepsilon}) = \mathrm{E}[(\hat{\varepsilon} - \varepsilon)^2]$, where for notational ease we denote $\varepsilon[\psi_n]$ and $\hat{\varepsilon}[\psi_n]$ by ε and $\hat{\varepsilon}$, respectively, or, equivalently, by the square root of the MSE, known as the *root-mean-square* (*RMS*). The expectation used here is relative to the sampling distribution induced by the feature-label distribution. The MSE is decomposed into the bias, $\mathrm{Bias}(\hat{\varepsilon}) = \mathrm{E}[\hat{\varepsilon} - \varepsilon]$, of the error estimator relative to the true error, and the deviation variance, $\mathrm{Var}_{\mathrm{dev}}(\hat{\varepsilon}) = \mathrm{Var}(\hat{\varepsilon} - \varepsilon)$, by

$$\mathrm{MSE}(\hat{\varepsilon}) = \mathrm{Var}_{\mathrm{dev}}(\hat{\varepsilon}) + \mathrm{Bias}(\hat{\varepsilon})^2. \tag{3}$$

III. Bayesian MMSE Error Estimator

Let $\pi(c)$, $\pi(\theta_0)$ and $\pi(\theta_1)$ denote the marginal priors of c, θ_0 and θ_1 respectively, and suppose data are used to find each posterior, $\pi^*(c)$, $\pi^*(\theta_0)$ and $\pi^*(\theta_1)$, respectively. Independence is preserved, i.e., $\pi^*(c, \theta_0, \theta_1) = \pi^*(c)\pi^*(\theta_0)\pi^*(\theta_1)$ [1]. If ψ_n is a trained classifier given by $\psi_n(\mathbf{x}) = 0$ if $\mathbf{x} \in R_0$ and $\psi_n(\mathbf{x}) = 1$ if $\mathbf{x} \in R_1$, where R_0 and R_1 are measurable sets partitioning the sample space, then the true error of ψ_n under the feature-label distribution parameterized by θ may be decomposed as

$$\begin{aligned} \varepsilon(\psi_n, \theta) &= c \int_{R_1} f_{\theta_0}(\mathbf{x}|0)\, d\mathbf{x} + (1-c) \int_{R_0} f_{\theta_1}(\mathbf{x}|1)\, d\mathbf{x} \\ &= c\varepsilon^0(\psi_n, \theta_0) + (1-c)\varepsilon^1(\psi_n, \theta_1), \end{aligned}$$

where $f_{\theta_y}(\mathbf{x}|y)$ is the class-y conditional density assuming parameter θ_y is true and ε^y is the error contributed by class y. The Bayesian MMSE error estimator can be expressed as [1]

$$\widehat{\varepsilon}(\psi_n, S_n) = \mathrm{E}_{\pi^*}[c]\mathrm{E}_{\pi^*}[\varepsilon^0] + (1 - \mathrm{E}_{\pi^*}[c])\mathrm{E}_{\pi^*}[\varepsilon^1]. \tag{4}$$

Given the sample and letting $\mathbf{\Theta}_y$ be the parameter space of θ_y,

$$\mathrm{E}_{\pi^*}[\varepsilon^y] = \int_{\mathbf{\Theta}_y} \varepsilon^y(\psi_n, \theta_y)\pi^*(\theta_y)d\theta_y. \tag{5}$$

The Bayesian MMSE error estimator can be found from *effective class-conditional densities*, which are derived by taking the expectations of the individual class-conditional densities with respect to the posterior distribution,

$$f\left(\mathbf{x}|y\right) = \int_{\mathbf{\Theta}_y} f_{\theta_y}\left(\mathbf{x}|y\right)\pi^*\left(\theta_y\right)d\theta_y. \tag{6}$$

Using these [2],

$$\widehat{\varepsilon}\left(\psi_n, S_n\right) = \mathrm{E}_{\pi^*}[c]\int_{R_1} f\left(\mathbf{x}|0\right)d\mathbf{x} + \left(1 - \mathrm{E}_{\pi^*}[c]\right)\int_{R_0} f\left(\mathbf{x}|1\right)d\mathbf{x}. \tag{7}$$

IV. Review of Literature Pertaining to Classification of Stochastic Processes

In the following we refer to “time” as being a generic term indicating an ordered set of indices. Pattern analysis of time-dependent data includes a wide variety of applications. Below are the most popular applications [3]:

i) Given a time series containing n points, predict the value at $n+1$ (*prediction*);

ii) Assign a set of unlabeled time series over time T to one of a family of pre-defined labeled time series over T (*classification*);

iii) Given a time series along with some similarity measures, find the most similar patterns in a collection of time series (*indexing*)

iv) Use some similarity measure to determine the “natural” grouping among a collection of times series (*clustering*)

In the current manuscript, we are concerned with a classification application and, therefore, in the subsequent discussion we will only focus on studies related to classification. Classification of time series data has different applications in various domains. For example, in seismology the classification of time-series data is used to classify earthquake data from data obtained from nuclear explosions [4], [5]. In engineering, an important application is to differentiate a signal generated by noise alone from a signal plus noise. In medicine this classification has been used to classify different stages of sleep by considering the contents of EEG signals [6], and to differentiate between levels of anesthesia that are sufficient for deep surgery [7]. There is quite a large body of work on discrimination of stochastic processes. In what follows we focus on several important achievements in the field and categorize them into several classes.

A. *Classical approach to discrimination of stochastic processes*

This line of work goes back to the early 1970's when Basu and Odell observed in remote sensing applications that the conditional expected true error of linear discriminant analysis (LDA) is commonly higher than what is expected from a theoretical analysis [8]. They associated this observation with violation of the independence assumption of training data.

To study the effect of correlated training data on the performance of LDA, Basu and Odell [8] used numerical examples under an equicorrelated structure of samples. They showed that misclassification probabilities change under such structures. McLachlan in [9] used asymptotic analysis to show that even under a simple-equicorrelated structure the probability of misclassification changes. Later, Tubbs [10] used a similar asymptotic analysis but with a serially correlated structure among training data. He considered further simplifying assumptions to show that the asymptotic error rate changes with serially correlated data having positive correlations. Lawoko and McLachlan [11] used the same serially correlated structure and obtained a different asymptotic expansion of LDA true error from the one that Tubbs previously had achieved in [10]. This type of asymptotic analysis was later used in [11], [12] to characterize the asymptotic expected true error of univariate LDA and Z-statistics assuming an autoregressive process of order p. In [13], we consider two general classes of Gaussian distributions under which we characterize the exact performance of LDA when the data are univariate. We show the application of the theory developed therein in situations where the data are generated from two autoregressive or two moving-average sources. Some work considers the problem of discrimination of time-series data by considering a Bayesian approach specific to univariate autoregressive processes [14], [15]. For example Broemeling and Son [14] consider the problem of assigning time-series data to an autoregressive source with unknown parameters. By considering a vague prior for unknown parameters, they train a model to assign an observed sample path to the class that maximizes the posterior mass function.

B. *Spectral approach to discrimination of stochastic processes*

Shumway and Unger [4] use the theory of discriminant analysis combined with spectral approximation and estimation for discriminating two Gaussian processes. In this regard, they derive the optimal discriminant in the sense of maximizing the Kullback-Leibler discrimination information rate, J-divergence rate, and detection probabilities. Then they use the Fourier transform to derive an approximation of the optimal discriminant based on spectral contents of stationary processes. At the end, they replace the unknown parameters appearing in the approximate discriminant by their sample estimates. In this work, the authors conclude that the benefit of using the spectral approximation is two fold: 1) the matrix

operations appearing in discrimination are replaced by simpler operations, including spectral and FFT; 2) more stable estimates are obtained from spectral estimation techniques than from covariance matrix estimation. For more detailed discussion of this work and its extension to non-Gaussian processes and clustering see [16].

The aforementioned work considers the problem of discrimination between stationary processes using parametric models. Yuan and Rao [17] consider classification of discrimination between two stationary processes using a non-parametric approach. They use a smoothed periodogram to estimate the spectral contents of the signals. The classification rule is then defined to be the classifier that minimizes the discrepancy of the periodogram from the class spectrum. The problem of discrimination between non-stationary (locally stationary) and non-Gaussian processes is considered in [18]. In this work the authors consider an approximation of the Gaussian Kullback-Leibler discrimination information rate as the classification statistic; that is, they compare the estimated rate to a pre-defined threshold and assign the labels.

C. Adaptive classification of stochastic processes

A more recently developed line of work focuses more on constructing classifiers in an adaptive setting. In this framework the classifier is updated on the arrival of new labeled data and, at the same time, the classifier becomes adapted to change of population over time, a concept commonly referred to as population drift. Bottcher et al. [19] construct and extrapolate a model of population drift in order to construct a decision tree for classifying a future sample point. In [20], Adams et al. use the concept of so-called “adaptive forgetting”. In short, a forgetting factor controls the amount of contribution of historical data–generally, the more recent the data, the more its contribution. This approach has been embedded in the theory of discriminant analysis (LDA and QDA) [20] to update the classifier using some recursion formulas for mean and covariance.

D. Semi-supervised classification of time-series

In [21] the authors study the effect of incorporating a large amount of unlabeled data to utilize in classification of time series data, hence, constructing a semi-supervised scheme. The semi-supervised classification of time-series is further studied in [22], [23].

REFERENCES

[1] L. Dalton and E. R. Dougherty, "Bayesian minimum mean-square error estimation for classification error–Part I: Definition and the Bayesian MMSE error estimator for discrete classification," *IEEE Trans. Sig. Proc.*, vol. 59, no. 1, pp. 115–129, 2011.

[2] ——, "Optimal classifiers with minimum expected error within a bayesian frameworkPart I: Discrete and gaussian models," *Pattern Recognition*, vol. 46, p. 13011314, 2013.

[3] C. A. Ratanamahatana, J. Lin, D. Gunopulos, E. Keogh, M. Vlachos, and G. Das, *Mining time series data. Data Mining and Knowledge Discovery Handbook*. Springer, 2005.

[4] R. H. Shumway and A. N. Unger, "Linear discriminant functions for stationary time series," *J. Am. Statist. Assoc.*, vol. 69, pp. 948–956, 1974.

[5] G. R. Dargahi-Nourbary, "Discrimination between gaussian time series based on their spectral differences," *Communications in Statistics*, vol. 21, pp. 2439–2458, 1992.

[6] J. Alagon, "Spectral discrimination for two groups of time series,," *J. Time Series Anal.*, vol. 10, pp. 203–214, 1989.

[7] W. Gersch, F. Martinelli, J. Yonemoto, M. D. Low, and J. A. MacEwan, "Automatic classification of electroencephalograms by k-l nearest neighbor rules," *Science*, vol. 205, p. 193195, 1979.

[8] J. P. Basu and P. L. Odell, "Effect of intraclass correlation among training samples on the misclassification probabilities of bayes' procedure," *Pattern Recogn.*, vol. 6, pp. 13–16, 1974.

[9] G. J. McLachlan, "Further results on the effect of interclass correlation among training samples in discriminant analysis," *Pattern Recogn.*, vol. 8, pp. 273–275, 1976.

[10] J. D. Tubbs, "Effect ofautocorrelated training samples on bayes' probability of misclasslficatlon," *Pattern Recogn.*, vol. 12, pp. 351–354, 1980.

[11] C. R. O. Lawoko and G. J. McLachlan, "Discrimination with autocorrelated observations," *Pattern Recogn.*, vol. 18, pp. 145–149, 1985.

[12] ——, "Asymptotic error rates of the w and z statistics when the training observations are dependent," *Pattern Recogn.*, vol. 19, pp. 467–471, 1986.

[13] A. Zollanvari and E. R. Dougherty, "Analytical study of performance of linear discriminant analysis in stochastic settings," *Pattern Recogn.*, vol. 46, pp. 3017–3029, 2013.

[14] L. D. Broemeling and M. S. Son, "The classification problem with autoregressive processes," *Commun. Statis. Theory Meth.*, vol. 16, pp. 927–936, 1987.

[15] V. R. Marco, D. M. Young, and D. W. Turner, "Predictive discrimination for autoregressive processes," *Pattern Recognition Letters*, vol. 25, pp. 145–149, 1988.

[16] Y. Kakizawa, R. Shumway, and M. Taniguchi, "Discrimination and clustering for multivariate time series," *J. Am. Statist. Assoc.*, vol. 93, pp. 328–340, 1998.

[17] J. Yuan and T. S. Rao, "Classification of textures using second-order spectra," *Science*, vol. 13, pp. 547–562, 1992.

[18] K. Sakiama and M. Taniguchi, "Discriminant analysis for locally stationary processes," *Journal of Multivariate Analysis*, vol. 90, pp. 282–300, 2004.

[19] M. Bottcher, M. Spott, and R. Kruse, "Predicting future decision trees from evolving data," *Proceedings of ICDM08*, pp. 33–42, 2008.

[20] N. M. Adams, D. K. Tasoulis, C. Anagnostopoulos, and D. Hand, "Temporally-adaptive linear classification for handeling population drift in credit scoring," *Proceedings of COMPSTAT10*, pp. 167–176, 2010.

[21] L. Wei and E. Keogh, “Semi-supervised time series classification,” *Proceedings of 23rd ICML*, pp. 1033–1040, 2006.

[22] C. A. Ratanamahatana and D. Wanichsan, “Stopping criterion selection for efficient semi-supervised time series classification,” *Software Engineering, Artificial Intelligence, Networking and Parallel/Distributed Computing*, vol. 149, pp. 1–14, 2008.

[23] K. Marussy and K. Buza, “Success: A new approach for semi-supervised classification of time-series,” *Artificial Intelligence and Soft Computing*, vol. 7894, p. 437447, 2015.
